# Chronology of Ksar Akil (Lebanon) and Implications for the Colonization of Europe by Anatomically Modern Humans

**DOI:** 10.1371/journal.pone.0072931

**Published:** 2013-09-11

**Authors:** Katerina Douka, Christopher A. Bergman, Robert E. M. Hedges, Frank P. Wesselingh, Thomas F. G. Higham

**Affiliations:** 1 Oxford Radiocarbon Accelerator Unit, Research Laboratory for Archaeology and the History of Art, University of Oxford, Oxford, United Kingdom; 2 URS Corporation, Cincinnati, Ohio, United States of America; 3 Naturalis Biodiversity Center, Leiden, The Netherlands; Institut de Biologia Evolutiva – Universitat Pompeu Fabra, Spain

## Abstract

The Out-of-Africa model holds that anatomically modern humans (AMH) evolved and dispersed from Africa into Asia, and later Europe. Palaeoanthropological evidence from the Near East assumes great importance, but AMH remains from the region are extremely scarce. ‘Egbert’, a now-lost AMH fossil from the key site of Ksar Akil (Lebanon) and ‘Ethelruda’, a recently re-discovered fragmentary maxilla from the same site, are two rare examples where human fossils are directly linked with early Upper Palaeolithic archaeological assemblages. Here we radiocarbon date the contexts from which Egbert and Ethelruda were recovered, as well as the levels above and below the findspots. In the absence of well-preserved organic materials, we primarily used marine shell beads, often regarded as indicative of behavioural modernity. Bayesian modelling allows for the construction of a chronostratigraphic framework for Ksar Akil, which supports several conclusions. The model-generated age estimates place Egbert between 40.8–39.2 ka cal BP (68.2% prob.) and Ethelruda between 42.4–41.7 ka cal BP (68.2% prob.). This indicates that Egbert is of an age comparable to that of the oldest directly-dated European AMH (Peştera cu Oase). Ethelruda is older, but on current estimates not older than the modern human teeth from Cavallo in Italy. The dating of the so-called “transitional” or Initial Upper Palaeolithic layers of the site may indicate that the passage from the Middle to Upper Palaeolithic at Ksar Akil, and possibly in the wider northern Levant, occurred later than previously estimated, casting some doubts on the assumed singular role of the region as a locus for human dispersals into Europe. Finally, tentative interpretations of the fossil's taxonomy, combined with the chronometric dating of Ethelruda's context, provides evidence that the transitional/IUP industries of Europe and the Levant, or at least some of them, may be the result of early modern human migration(s).

## Introduction

Significant changes in human behaviour, cognition and innovation become sharply evident in the archaeological record of Eurasia at 45,000 years BP and demarcate the end of the Middle Palaeolithic and the onset of the Upper Palaeolithic period. The material cultures associated with the latter include the so-called “transitional” technocomplexes (e.g., the Châtelperronian of Franco-Cantabria, the Uluzzian of Italy and the Bachokirian of Bulgaria), and the subsequent Early Upper Palaeolithic (EUP) technocomplexes, namely the (Proto- and Early) Aurignacian found throughout the continent. In the Eastern Mediterranean region (hereafter, the Levant) the earliest Upper Palaeolithic includes the Emiran and Initial Upper Palaeolithic (IUP) entities, and the succeeding EUP, locally known as the Early Ahmarian, technocomplex. When compared to the Middle Palaeolithic record, these technocomplexes exhibit technological and typological diversification in stone tools made on blades, occasional production of organic implements from bone and antler, and importantly, the sudden appearance of personal ornamentation in the form of marine shell beads. These were not part of the behavioural package of previous human populations (Neanderthals) living in the same region.

Transitional/IUP and EUP assemblages, both in Europe and the Levant, have been attributed to the expansion of AMH and the replacement of local Neanderthal populations [1]–[3], although there are widely acknowledged limitations expressed in these linkages [4], [5], especially since there is such scanty fossil evidence in association. In addition to being extremely rare, human fossils from the period are usually fragmentary and difficult to characterize morphologically with certainty [6], [7], in some cases they lack an archaeological context (e.g., as at Peştera cu Oase), and/or are intrusive and of much younger (Holocene) age [8], [9].

Recently, the initial colonization of Europe by AMH has been shown to be earlier than previously thought, dating to ∼43–45,000 BP, or even earlier [10], [11]. This early presence, along with subsequent and probably more substantial movements of AMH towards Europe, e.g., during the Aurignacian, are thought to have occurred along two different trajectories, both starting or passing through the Near East [2], [3] and following a path either along the Mediterranean rim and/or up the Danube fluvial corridor.

The Levant represents a land bridge connecting Africa, Asia and Europe [12] and has often been viewed as a region of high palaeoanthropological significance, a starting point where one might expect to find some of the earliest AMH fossils alongside EUP assemblages [13], and possibly IUP assemblages, as well. Indeed, important examples of human fossils in such contexts were recovered 75 years ago at Ksar Akil in Lebanon. The site and the fossils, however, lacked a secure absolute chronology, which is the focus of the present paper. We believe these data may help further our understanding of the timing and geographic context of the dispersal of AMH into Europe.

### Context

**Figure 1:**
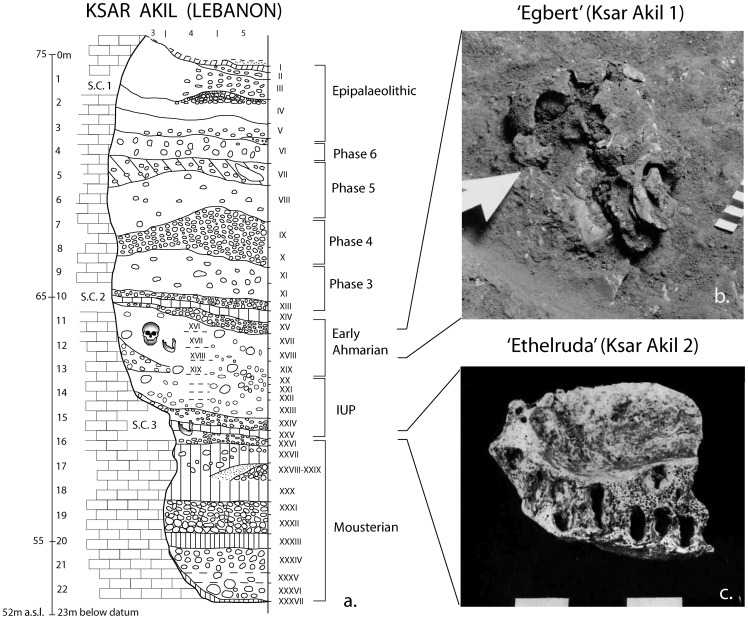
Stratigraphic and photographic documentation of the Ksar Akil excavations. (a) Stratigraphic sequence as established by the early excavations of Boston College. The section drawing of the 23 m-deep stratigraphy illustrates both the archaeological levels (in Latin numerals) and the broad techno-typologically distinct phases (Mousterian, Initial Upper Palaeolithic, etc.) that these levels have been ascribed to; (b) The discovery of Egbert (Ksar Akil 1) in 1938. Close up of the skull *in situ*. Image copyright Pitt Rivers Museum, University of Oxford (accession number: 1998.294.820); (c) Inferior view of the partial right maxilla of Ethelruda (Ksar Akil 2), modified after [32].

Ksar Akil is the reference Upper Palaeolithic site for the Near East. It was excavated by the American Jesuits Doherty, Ewing, and Murphy in 1937–38 and 1947–48 [14], [15], and later by Tixier between 1969–1975 [16] (Text S1 (*SI Section I)*). It contains a 23 m stratigraphic succession traditionally divided into 36 levels, I-XXXVI from top to bottom (Fig. 1; see also Text S1 (*SI Section I*)). During the most recent excavations by Tixier, many more levels and sub-divisions were established due to more advanced and thorough recovery procedures. Unfortunately, Tixier's excavations stopped before ever reaching the important IUP and EUP levels, due to political instability in Lebanon in the mid-1970s.

The earliest occupation at Ksar Akil, from level XXXVI to level XXVI at the base of the sequence, is of Middle Palaeolithic (Mousterian) affinities ([17]–[18]; see also Text S1 (*SI Section I*)). The sequence continues with an intermediate archaeological phase, which represents the transition from the Middle to Upper Palaeolithic period. Assemblages exhibiting similar characteristics are currently referred to as IUP [19]–[20]. At Ksar Akil, the IUP phase occupies levels XXV–XXI ([21]–[22]; see also Text S1 *(SI Section I)*). The subsequent archaeological levels XX–XVI display a shift in some of the characteristics of material culture and lithic assemblages towards a classic Upper Palaeolithic manifestation, known as the Ahmarian, specifically the Northern facies of the Early Ahmarian ([23]; see also Text S1 *(SI Section I*)). Levels XV and XIV contain little evidence for human presence and are thought to represent an occupational hiatus, possibly reflecting an episode of intensive soil weathering during a wet climatic phase [14], [17]. The upper portion of the Ksar Akil sequence comprises levels XIII–VI that span approximately 7.25 meters of deposit. Previously referred to as Levantine Aurignacian A, B, and C [24], recent studies [25]–[29] have avoided this descriptor due to a lack of clarity surrounding what constitutes the Aurignacian presence in the Levant. This upper portion of the Ksar Akil sequence is stratigraphically and culturally complex and is summarized in Text S1 (*Section II),* Table S1. It is very likely that the lower portion is equally complex; however, detailed studies have not been undertaken yet.

An interesting feature in the long stratigraphy of the site is the presence of three, well-defined geological formations referred to as ‘Stone Complex’ 1, 2 and 3. These are tripartite layers of cemented angular stones separated by sterile red clay (an *in situ* soil formation, product of limestone weathering) found at 1.5 m, 10 m and 15 m below datum, respectively. They are traditionally interpreted as indications of a significant environmental instability, e.g., increased precipitation during a wet phase, affecting the pedostratigraphy of the site.

### Ksar Akil 1: *‘Egbert’*

In 1938, Doherty's team discovered the skull and postcranial remains of a juvenile *Homo sapiens* referred to as Ksar Akil 1, and more commonly known as “Egbert” [14]–[15], [30]. The remains were found close to the rockshelter wall, 11.46 m below datum in square F 3, in level XVII (or XVIII). These levels are associated with the Early Ahmarian, a classic Upper Palaeolithic industry in the Levant. Egbert was covered by a pile of water-worn boulders, which seem to indicate deliberate internment. An additional maxilla and some rib fragments were found very close to the body, indicating that a second individual may have been buried at the same location. The fossils were poorly preserved and mostly encased in breccia [30]. Only the skull was extracted and reconstructed [31]. A metre above the burial(s), a break in the geological and cultural sequence, a cemented formation referred to as Stone Complex 2, separates the Early Ahmarian of levels XX–XVI from the subsequent later Upper Palaeolithic levels XIII–VI [14], [21]–[22].

The Egbert fossil is currently known only from descriptions, photographs and reconstructed casts of the skull. Based on the British Museum casts EM 274 and EM 275, Bergman and Stringer [30] confirmed Ewing's initial assessment [14], [15] that Egbert is an anatomically modern specimen belonging to a young individual, possibly female, of about 7–9 years age at death [30]. The cranium is small and delicately built and there is no visible supraorbital torus development. The zygomatic and maxillary areas, as well as the vault and the mandible, reveal an anatomically modern shape [30].

### Ksar Akil 2: ‘Ethelruda’

During the second field campaign at Ksar Akil, between 1947 and 1948, a partial maxilla was recovered from a level stratigraphically deeper than Egbert. Ksar Akil 2 (referred to as ‘Ethelruda’) was found in level XXV, three meters from the face of the cliff, 15 m below datum. Level XXV was described as a red clay, part of the lowest Stone Complex 3 and Ewing [32], [33] noted that the maxilla was “definitely associated with an important change in geology and lithic tradition”, now understood to represent the start of the IUP, the transitional industry of northern Levant ([20]; see also Text S1 (*SI Section I*)). Interestingly, a single atypical Emireh point from Ksar Akil, the *fossile directeur* for the industry, was also discovered in level XXV. Ethelruda was thought to be lost for many years, but the fossil has recently been located in storage at the National Museum in Beirut (Directorate General of Antiquities in Lebanon, Serial Number: 25724; see [34]).

The fossil consists of part of the right maxilla, and a small portion of the left. It lacks all teeth, with the exception of the right canine root [32]. Ewing [32] attributed the specimen to a “Neandertaloid” female adult on the basis of comparative metric analysis with the fossils from Tabun I, Skhul IV and V, Gibraltar and Chapelle-aux-Saints. This attribution however has been questioned in recent years. According to Metni [35], the published measurements of the maxilla fall within anatomically modern ranges, which Ewing did not consider in his study. In addition, Ewing reported that the Ethelruda fossil was morphologically similar to Skhul V, which at the time was thought to be a Neanderthal. Currently, Skhul V is considered to be an archaic form of modern human [36], dating to between 110–90 ka [37] or later [38]. Metni [35], Copeland and Yazbeck [34] and Yazbeck [39] have suggested, therefore, that Ethelruda may be an anatomically modern human. Further analytical work on the fossil is planned (C. Stringer pers. comm.) and this will finalize the taxonomic status of the fossil.

With the exception of about 10 teeth from the IUP and Early Ahmarian levels of Üçağızlı Cave in southern Turkey, most likely belonging to *Homo sapiens* [40]–[41], Egbert and Ethelruda are the only other human fossils in the Near East directly linked to EUP and IUP assemblages, respectively.

The lack of a firm chronostratigraphic framework for the Ksar Akil sequence and the fact that neither the IUP nor the Early Ahmarian levels of the site have been dated before, means that great deal of uncertainty surrounds the age of the fossils and their contexts. Their relationship, whether ancestral, contemporaneous or descendant, to the Upper Palaeolithic European technocomplexes and to other EUP humans of Eurasia and Africa remains unknown.

## Materials and Dating Methods

Initial attempts to date bone material from Ksar Akil were unsuccessful due to the complete absence of collagen (Text S1 (*SI Section II*)). Both faunal remains, as well as modified bone objects and tools, the latter sampled by our team in 2008 at the University of Bordeaux (Inv. numbers: KA-73/9, KA-72/62, KA-74/59, KA-72/43, KA-75/49, KA-74/26, KA-75/69, KA-72/42, KA-72/55, KA-70/72–73; with the permission of Prof. Fr. d'Errico), preserved no organics. Since no charcoal was available from Ksar Akil, another type of material was required for dating purposes.

In the late Middle Palaeolithic, but mainly during the Upper Palaeolithic periods, marine shell was regularly transported to the site from about 6–10 km away for use as personal ornaments, tools or for food [42]–[44]. The molluscan collection from Ksar Akil is in fact one of the largest ever discovered at a Palaeolithic site. It contains impressive numbers of both marine and terrestrial snails (∼2000 specimens), the vast majority of which carry evidence of human modification such as perforation for suspension, burning, polishing, snapping, and ochre residues.

Recent research into ways of addressing contamination when dating shell carbonates [45] has led to the development of a new pre-treatment and pre-screening protocol [46], which was used here to date the Ksar Akil marine shells. This research [45] has also identified that the selection of “aged” gastropod shells was very limited during the Upper Palaeolithic period, possibly due to the poor quality of weathered marine shell for piercing and manufacture into beads. We, therefore, consider the age of the shell material used for beads to be closely related in the majority of the cases to its selection and use by humans, as well as its subsequent deposition at the site. No ornaments were found in direct association with the human remains from Ksar Akil despite the fact that large numbers of shell beads were discovered a few centimetres above and below Egbert's location, in levels XVII and XVIII. Some of these beads were radiocarbon dated and are used below to statistically constrain the age of the fossil.

Radiocarbon dating was performed at the Oxford Radiocarbon Accelerator Unit (ORAU). The methods employed in dating shell and charcoal material in the ORAU have been reviewed by Douka et al. [45], [46] and Brock et al. [47], respectively. Overall, we dated 26 shells from Ewing's levels XXVII–IX obtaining 30 new AMS dates (Text S1 (*SI Section II*), Table S3). All dated specimens were located at the Naturalis Natural History Museum (Leiden, The Netherlands) and their inventory numbers are: RGM 550233, 550238, 550219, 550220, 550221, 550223, 550215, 550198, 550200, 550216, 550226, 550197, 550225, 550195, 550227, 550222, 550196, 550228, 550230, 550199, 550231, 550232, 550236. All necessary permits were obtained for the described study, which complied with all relevant regulations.

Of the sampled material, twenty-one dated gastropod shells had been transformed into beads, while five were of bivalves with evidence of human manipulation (Fig. S2). The dated samples were generally well preserved and no major mineral substitutions were observed after thorough screening of the carbonate matrix using X-Ray diffraction.

One charcoal sample from Tixier's excavations, dated in Oxford in the late 1980s using a less-refined method (ABA), was subjected to a harsher pre-cleaning protocol (ABOx-SC). This protocol has been shown to provide more reliable results, especially for old (>30 ka BP) charcoal [48]–[49]. All radiocarbon determinations were calibrated using the IntCal-Marine09 curve [50] on the OxCal 4.1.7 software [51]. Bayesian statistical methods were employed to analyse the results [51] (Text S1 (*SI Section III*), Figs. S4–S5). Comparisons to the NGRIP δ^18^O record [52] were used for broad climatic correlations.

## Results

The new AMS dates on shell range from 39.5 ka BP for the late Mousterian level XXVIII to ∼30–29 ka BP for level VIII (Text S1 (*SI Section II*), Table S3 *)*. An additional measurement on charcoal, dated previously at the ORAU at 29.3±0.8 ka BP, was re-dated after pre-treatment with a more rigorous protocol (ABOx-SC) at 30.2±0.17 ka BP. The new date is statistically identical, but has a significantly higher measurement precision, and should be considered more reliable.

Bayesian methods allow the formal incorporation, along with the calibrated radiocarbon likelihoods, of all lines of evidence pertaining to the chronostratigraphy of a site, such as breaks in the sequence and the succession of archaeological levels. In the case of Ksar Akil, two Bayesian models were built to account for the degree of variation observed in the radiocarbon results, as well as the uncertainties regarding the attribution of shell beads to particular levels.

Initially, most of the old and all of the new determinations were incorporated in a model structured around the individual levels from which the dated samples derive. In the second model, the individual levels were combined within five broad techno-typologically distinct phases and the results were grouped in them; the assigned depth for each shell was not taken into account since it only refers to the top of each level (often 1–2 m thick) and not the actual position of the shell therein (for further model specifications see Text S1, (*SI Section III)*). The second model is more flexible and allows for a certain degree of material movement through levels found in close proximity. It also incorporates most available data with less statistical outliers. It should be pointed out that the stratigraphy is defined in geological layers that did not necessarily align with the archaeological levels and their accompanying artifacts. In addition, several closely associated levels (e.g. XVIII–XVII–XVI), often indistinguishable in the field, are regarded as being developmentally very closely related. Modelling these levels as a single “Phase”, therefore, does not distort the archaeological association of the material.

### Bayesian modelling output

**Figure 2:**
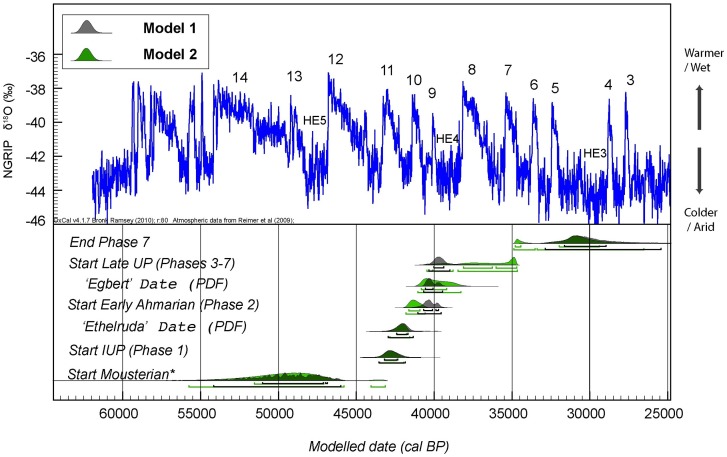
Comparison of the start boundaries for each archaeological phase, produced by the two Bayesian models for Ksar Akil (shown in Text S1 (*SI Section III), Figs. S4–5* *)*. The boundaries, undated events, reflect the most likely age for the beginning of each of the major technocomplexes. Models are shown in different colours. The only area of significant discrepancy is the start boundary of the late Upper Palaeolithic phase (Phase 3), starting with layer XIII directly following Stone Complex 2. The start boundary for the Mousterian is very tentative, since there are no determinations from the lowermost part of the Mousterian phase. The boundaries and PDFs are compared to the NGRIP δ^18^O record [52] and the Greenland Interstadials are numbered, as are the two relevant Heinrich Events 3 and 4.

The output of the two models described above is very similar (Fig. 2). Eleven outliers are identified in Model 1 (∼28%, Fig. S4) and 9 in Model 2 (∼23%, Fig. S5). This is higher than expected through statistical variation alone. One species of shell, *Columbella rustica*, gave consistently variable results and may be seriously affected by post-excavation mixing (Text S1, *SI Section III*). Excluding determinations of this species, the number of outliers in Model 2 drops to about 12%.

The age of the basal part of the Ksar Akil sequence is effectively unknown and probably greater than 50 ka BP. According to the modelling output (both iterations), the Mousterian terminates at 43.2–42.4 ka cal BP (68.2% confidence level) and is followed by the IUP (Ksar Akil Phase 1 of Ohnuma and Bergman). No dates exist for the lowermost IUP levels XXV–XXIV; the phase appears to be brief and lasts until about 41.6–40.9 ka cal BP (68.2%; Model 2), or a millennium later based on Model 1, when the Early Ahmarian begins. The largest difference in the modelling output concerns the end of the Ahmarian and the start of the later Upper Palaeolithic phases (Phases 3–6 of Williams and Bergman). In the first scenario (Model 1), the end boundary of the Early Ahmarian is estimated at ∼40.1–39.5 ka cal BP, while in the second (Model 2) it is at 39–37.5 ka cal BP (Fig. 2). Stratigraphically, the Early Ahmarian is succeeded by sterile level XIV and Stone Complex 2. Interestingly, the modelled span for the formation of Stone Complex 2 coincides in both cases with the climate deterioration during Heinrich Event 4, centred around 40 and 38 ka cal BP (Fig. 2 and Figs. S4–S5). This confirms the assessment of early geologists [19] that this geological formation represents a period of significant climatic variability. Ksar Akil Phase 3 starts immediately after, in Model 1 at 40.0–39.3 ka cal BP and in Model 2 at 38.1–34.6 ka cal BP (Fig. 2 and Figs. S4–S5).

We used the “Date” function of OxCal to calculate a probability distribution function (PDF) for the fossils' likely age within the modelled sequence. The determinations from the Early Ahmarian layers XVII and XVIII, where Egbert was found, as well as the determinations from above and below these layers constrain the probable age of the specimen. In addition, the modelled age for the beginning of Stone Complex 2 provides a *terminus ante quem* for the deposition of the fossil. This PDF is based on the assumption that Egbert was excavated in its original location and is not intrusive from much higher levels. We believe this to be true based on photographic documentation and other lines of evidence. For example, there was no visible pit to suggest downwards intrusion from a much higher level, and part of the body was found protected by large water-worn and carefully placed boulders, which may suggest that the child was buried at about the same level as the occupation floor. The boulders covering the body at the back of the rockshelter limited major post-depositional disturbances, as did the presence of intact Stone Complex 2 directly above the burial.

The same can be claimed for Ethelruda, discovered within the middle clay layer of Stone Complex 3, and covered by a layer of angular limestone flakes [32]. Ewing initially claimed that the maxilla belonged to level XXIV, on the assumption that XXV was sterile and a purely geological stratum [32]. In 1966 however, he published a corrigendum [33] where he acknowledges that following Hooijer's study [53], level XXV was not sterile, but instead contained substantial amounts of faunal remains [33] as well as evidence for hearths (D. Garrod photographic archive). Consequently, the stratigraphic position of Ethelruda was reinstated to level XXV [53]. In the Bayesian model, the determinations from the uppermost Mousterian levels, as well as the ones from the IUP were used to constrain the age of the fossil.

**Figure 3:**
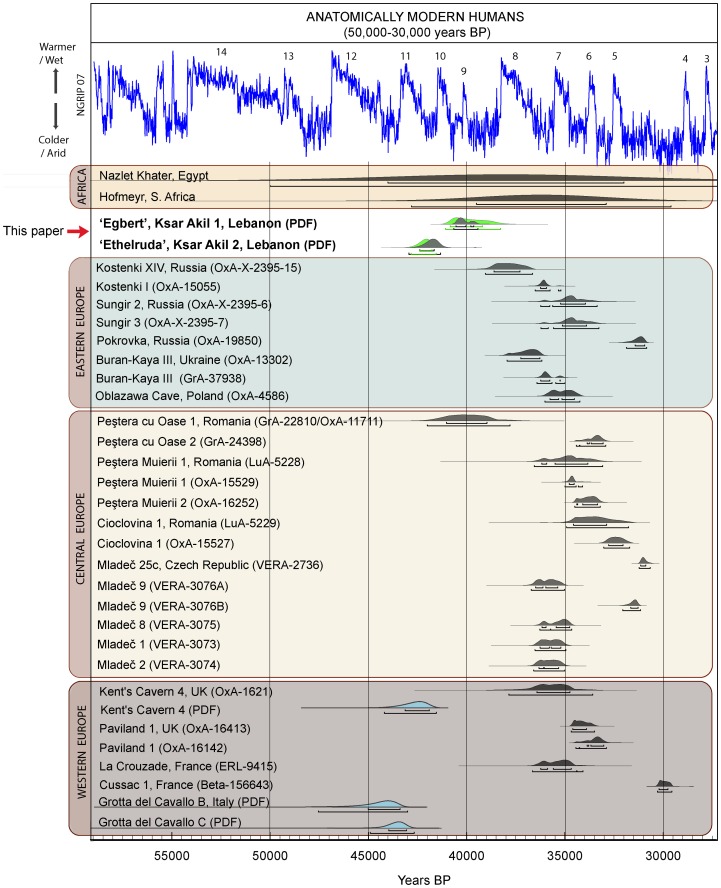
Comparison of the modelled ages (*Probability Distribution Function; PDF*) obtained for Egbert and Ethelruda with age estimates of AMH from other Palaeolithic sites between 50,000–30,000 years ago. The PDFs for the Ksar Akil fossils (Model 1 in green, Model 2 in black), as derived from the Bayesian modelling (Text S1 (*SI Section III), Figs. S4–5*), are plotted against the currently available determinations for AMH from Europe and Africa [9], [10]. The likelihoods for the directly-dated specimens are shown in dark grey, whereas the PDFs for those dated indirectly, in light blue. Egbert is contemporaneous with the oldest directly-dated European modern human (Peştera cu Oase, [54]) and falls within the earlier part of the ranges for both Nazlet Khater and Hofmeyer, the African AMH; these dates, however, are very imprecise. The Peştera cu Oase date is a mean of two determinations, one ultrafiltered and one not. The age estimate for Ethelruda is broadly similar to that for the AMH maxilla from Kent's Cavern, but not older than the AMH teeth from Cavallo in Italy. The radiocarbon determinations were calibrated with the INTCAL09/ Marine09 curve [50] and the modelling was performed using OxCal v.4.1.7 [51].

The calculated PDF for the age of Egbert corresponds to 40,850–39,200 cal BP (68.2% prob.) or 41,050–38,300 cal BP (95.4% prob.) (Figs. 2–3). We have run more than 10 variations of Bayesian models to assess the sensitivity of our modelled results. In these, the priors were slightly changed, e.g., determinations were grouped, un-grouped, moved across contexts or completely excluded, and in all cases the particular PDF falls sharply between 41–39 ka cal BP. Our conclusion is that the age estimate for Egbert is robust.

For the oldest specimen, Ethelruda, the calculated PDF is earlier and corresponds to 42,400–41,750 cal BP (68.2% prob.) or 42,850–41,550 cal BP (95.4% prob.). The results from both models presented above are identical. In the case of Ethelruda, our estimate is constrained by only a small number of determinations above and below the fossil. We acknowledge that the addition of further measurements may alter this estimate, possibly towards earlier dates. For the moment, however, this is the most reliable age estimate for this fossil.

## Discussion

### Comparison of the Ksar Akil specimens with directly and indirectly dated modern humans

In the past three decades, several AMH remains from across Eurasia and Africa have been directly dated between 50–30 ka BP, although many of these determinations are potentially problematic due to insufficient decontamination methods used in the past. Only a small number of human bones have recently been directly dated using up-to-date methodologies; these include the skeletal remains from Kostenki 1 and Kostenki 14, Sungir 2 and 3, Pokrovka, Peştera cu Oase and Peştera Muierii, Cioclovina, Buran Kaya (partially) and Paviland (see references in [9]–[10]).

Important new evidence for the early presence of modern humans in Europe has been generated in recent years through indirect dating of human fossils using methods identical to those employed here, namely Bayesian statistical modelling of chronometric determinations and PDFs. A modern human maxilla fragment (KC4) from Kent's Cavern in Great Britain, associated with only a very small number of chronologically non-diagnostic implements, is placed between 44,200–41,500 cal BP [10]. In Italy, two deciduous molars from the transitional Uluzzian levels of Grotta del Cavallo were ascribed to AMH, and not Neanderthals as originally thought, and on the basis of direct radiocarbon dating of shell beads from the same layers, the PDF for the most likely age of the teeth was determined to be 45,000–43,000 cal BP, and possibly earlier [11]. Currently these are the oldest, indirectly dated, fossils in Europe.

Comparison of the PDF generated here for the Ksar Akil specimens with the PDFs for the indirectly dated AMH fossils from Cavallo and Kent's Cavern, as well as with radiometric ages of the directly dated European and African AMH, allows for an overall assessment of their antiquity and inferred phylogenetic relationships with these human fossils (Fig. 3). The PDFs for Cavallo and Kent's Cavern [10], [11] clearly predate that of Egbert. Interestingly, the age estimate for Egbert overlaps significantly with the oldest directly-dated AMH fossil in Europe, the Peştera cu Oase mandible from Romania [54] (Fig. 3). Unfortunately, the Peştera cu Oase remains are devoid of an archaeological or cultural affiliation. The age estimate for Egbert is in accordance with current dating evidence for the appearance of the fully Upper Palaeolithic technocomplexes in Europe [55], [56], widely accepted as the result of the establishment of AMH populations on the continent.

Ethelruda is older and its age appears close to that of contexts where Cavallo C and KC4 were recovered. It is worth noting that, just as with the Ksar Akil specimen, Cavallo C also comes from a transitional (Uluzzian) context.

The vast majority of the remaining directly dated modern human remains (Fig. 3) are considerably younger. However, as previously noted, only a few were dated with state-of-the-art methodologies. Constant improvements in the methods used for the dating of old and potentially contaminated samples, and their application to such specimens (e.g. [57]), are needed if we are to verify their age.

### The Levant as a dispersal route

Egbert is clearly not associated with the onset of the Upper Palaeolithic at Ksar Akil, but Ethelruda appears to be. The determinations we have obtained from the site for the beginning of the IUP at around 41–42 ka cal BP correspond well with those of other, recently-dated transitional industries in Europe, but they are not older than these. This observation has potential implications for the position of the Levant during the Middle to Upper Palaeolithic transition and in the process of the colonisation of Europe by modern humans.

Despite increasing archaeological and genetic data in support of an African origin for modern humans, there is little consensus about the exact timing or about the route or routes taken during migration(s) out of Africa and into Asia and Europe [58]. The notion that modern humans dispersed first into the Near East and then directly into Europe, is a common perception amongst palaeoanthropologists and prehistorians. It is currently accepted that modern humans migrated from Africa in several waves, probably using a number of dispersal routes. Garcea [59] distinguishes two Out of Africa movements by AMH, on the basis of individual features and of being separated by a long time span. The earlier wave or “Out of Africa 2a” took place between about 130,000 and 80,000 years ago, while the second “Out of Africa 2b” occurred at ∼50,000 years ago, after an apparent gap of about 30,000 years [59], [60].

In the Levant, the archaic modern humans from Qafzeh and Skhul, manufacturing Middle Palaeolithic tools and dating to between 130–80 ka BP [37] (but see [38]), form the basis of an early exodus of modern humans through the region. Spatially explicit modeling of the expansion of AMH allied with climate reconstructions over the past 120 kyr [61] suggests that population movement mainly occurred along the southern route, crossing into the Arabian Peninsula at its most southerly point. Other research supports this conclusion, (e.g. [62]). However, it is thought that this early modern human genetic lineage became extinct, possibly at the transition from MIS 5a to MIS 4 (∼74,000 years ago) and did not contribute to the much later AMH colonization of Europe [63]. What occurs therefore tens of millennia after the early expansion and the details of the second (re-)population of the Levant by AMH, remains unclear and the archaeological record of the region is at best sparse and difficult to interpret.

Following Qafzeh and Skull, all other fossils that have been recovered in the Levant belong to Neanderthals until the time of Ethelruda and the likely-modern teeth from Üçağızlı [40], [41], to be followed later by Egbert. The last two cases, and Ethelruda if proved to be a modern human, may be seen as representatives of the second wave of AMH human expansion in the region (Garcea's “Out of Africa 2b”). By then, fully anatomically and behaviourally modern human groups appear to possess an Upper Palaeolithic toolkit identified with the IUP and EUP technocomplexes.

Dating evidence from IUP and EUP sites in the wider Near East, such as Üçağızlı Cave in Turkey may be compared with our new results from Ksar Akil. Based on the currently published dates [40] and the output of Bayesian modelling, the IUP (layers I–F) in Üçağızlı starts between 44.3–43.5 ka cal BP (68.2%) and the Early Ahmarian (layers E (?)–B) starts around 41.6–40.3 ka cal BP (68.2%). The IUP in Üçağızlı seems to precede that of Ksar Akil by 1–2 millennia, despite significant similarities between the lithic assemblages. It should be remembered, however, that the lowermost IUP levels (XXV–XXIV) in Ksar Akil have not been directly dated and there is a wide degree of chronological overlap between the IUP at both sites. The Early Ahmarian is roughly contemporaneous at both Ksar Akil and Üçağızlı.

In Umm el Tlel (Syria), levels III2a' and IIbase, described as “Paléolithique intermédiaire”, have been dated rather later, at 36.5±2.5 ka by TL on burnt flint, and at 34.5±0.89 ka BP with AMS dating [64].

Much earlier dates, however, are often cited for the beginning of the IUP in southern Levant, all from the open-air site of Boker Tachtit [65]. There, basal level 1 is associated with four conventional radiocarbon determinations on charcoal (SMU-580: 47284±9048, SMU-259: 46930±2420, SMU-184>45570, GY-3642>34950 BP). These ages were produced almost 30 years ago with old pre-treatment protocols and are very imprecise, possibly reflecting the low amount of carbon in the dated samples, an indication of possible sample heterogeneity. No effort has been made to reproduce or add to these original dates from Boker Tachtit, which have become central in the discussion of the early arrival of AMH in the Levant.

Another set of early charcoal dates from Kebara Cave [13], [66] place the start of the EUP, specifically the Early Ahmarian Unit IV, at ∼48–46 ka cal BP. Kebara is currently the only site where such early determinations have been obtained for a classic Upper Palaeolithic assemblage. The site, unlike Ksar Akil and Üçağızlı, lacks an IUP layer between the late Middle Palaeolithic Unit V and the Early Ahmarian Unit IV, but instead an unconformity is present at this interface. Complex site-formation processes and erosion-induced sloping surfaces at the interface of these adjacent stratigraphic units, as well as burrows and a large channel cutting through the Middle Palaeolithic and Early Ahmarian units [13], [66], render the association of the dated charcoals with the archaeology they are thought to date potentially problematic. Some inconsistencies observed between charcoal determinations produced with the routine (ABA) and more rigorous (ABOx-SC) pretreatment methods [66] may be suggestive of: (i) complex chemical processes affecting the samples and/or; (ii) charcoal groups of different ages existing within adjacent units V and IV. Rebollo et al. [66] state “*assuming that in the future such [early] dates will be supported by samples from Ksar Akil, the Levantine IUP and EUP assemblages herald the diffusion of UP technologies into Europe*.” Ksar Akil with its long record of Upper Palaeolithic occupation, and Üçağızlı, have both yielded determinations that are considerably younger, by at least 3–5 millennia, and do not support such a simple unidirectional model of cultural and/or demic diffusion. Clearly more work is needed to determine whether the Early Ahmarian dates from Kebara, as well as those from the IUP layers of Boker Tachtit, are accurate and can be corroborated by data from nearby sites.

The new chronometric results and Bayesian model from the reference Palaeolithic site of Ksar Akil suggest that: (i) both the IUP and the EUP of the northern Levant are roughly contemporaneous with, and not older than, their corresponding (transitional and Proto- or Early Aurignacian) technocomplexes in Europe and; (ii) neither Ethelruda nor Egbert are ancestral to European fossils associated with transitional and classic Upper Palaeolithic contexts, respectively.

While the Levant appears an obvious route in and out of Africa based solely on its geographical position, there is, as yet, no evidence for significant human and animal migrations during the Pleistocene ([67], and papers therein), let alone during the short time window of the Middle to Upper Palaeolithic transition or during the “Out of Africa 2b” scenario. The current evidence for contemporaneity in the appearance of both transitional/IUP and EUP technocomplexes in Europe and the Levant implies that the northern Mediterranean Levantine coast might not be the point of origin for the dispersal of the earliest Upper Palaeolithic outwards and into Europe. This, in turn, suggests to us that current models based on old assumptions regarding the pathway(s) of human dispersal require further testing and, possibly, revision.

### The makers of the IUP

The Levantine late Middle Palaeolithic is solely associated with Neanderthals [68], and Ewing's attribution of Ethelruda to a “Neandertaloid” individual has helped shaping a generalized view that Neanderthals may have been involved in the making of the transitional/IUP layers, at least in its earliest phases (e.g. [5]). The most recent archaeological and palaeoanthropological data from Ksar Akil with regards Ethelruda (e.g. [35]), as well as the likely-modern teeth from Üçağızlı, where Mousterian levels have not been identified, may provide a contrary view to traditional assumptions on the authorship of the IUP by Neanderthals. Of course, it is wise to remain cautious on the taxonomic status of both Ethelruda and the teeth from Üçağızlı until more detailed scientific work has been undertaken using state-of the-art methodologies. Further work is also urgently required to address whether other technocomplexes exhibiting similar characteristics, such as the Balkan Bachokirian (e.g. level 11 of the eponymous site, containing fossil remains; [69]) or the Bohunician (or Emiro-Bohunician; [70]) of Central Europe, fall into the same category.

## Supporting Information

Figure S1 **Location of the Ksar Akil site, Lebanon, a few kms NE of Beirut.**
(TIF)

Figure S2 **Examples of the dated shell specimens from Ksar Akil.** The vast majority consists of beads of *Nassarius gibbosulus/ circumcinctus* while KA 30 is an example of *Columbella rustica* shell. KA 54, an *Ostrea* sp. shell, is one of the very few shells coming from Middle Palaeolithic layers.
(TIF)

Figure S3 **Plot of all available dates from square E4.** OxA-20491 and OxA-25656 come from adjacent square (F5) and are used here as a *terminus post quem*. The determinations are plotted here together in order to check the chronological variation among specimens deriving from the same excavation square. The dates are consistent with the stratigraphic position. Assuming constant sedimentation (an over-simplified scenario), we may calculate an accumulation rate of 0.88 m of sediment deposited at the site every 1000 years.
(TIF)

Figure S4 **Bayesian Model 1.** Initial Bayesian plot with all new dates, as well as previously obtained ones from the Tixier excavations. The model is structured around individual layers and phases. Of the 39 determinations, 11 are flagged as outliers.
(TIFF)

Figure S5 **Bayesian Model 2.** Second modeling iteration containing most available dates from the Early Upper and Middle Palaeolithic levels of the site, including previously obtained dates. Here, individual layers are grouped together within broad industrial phases (see text for details). Of the 39 determinations, 9 outliers are identified.
(TIFF)

Table S1 **Archeological correlation of the Ksar Akil sequence, Boston College excavations (1937–1938, 1947–1948) and Tixier excavations (1969–1975).**
(DOC)

Table S2 **Previous chronometric determinations from Ksar Akil.** The majority of the dates relate to the late Upper Palaeolithic layers and were obtained on material from Tixier's excavation.
(DOC)

Table S3 **New radiocarbon determinations from Ksar Akil and details for stratigraphic details for each sample.** KA 51 was dated twice as it underwent mineralogical separation (see [32]) due to the presence of calcite in the original fraction. In the last column the percentage of secondary calcite in the shell matrix, established by XRD analysis, is indicated. The differentiation between *Nassarius gibbosulus* or *Nassarius circumcinctus* was not always possible due to the preservation state of the shells; here they are all tentatively ascribed to the former species. The δ^13^C value is also given when this was unusual for marine shells, therefore indicating either some degree of meteoric diagenesis or other technical issues. The 3 determinations marked with an asterisk were not used in the modeling since they are most certainly problematic (see text).
(DOC)

Text S1. (DOC)
