# Supplementary figures and images for: Chronology of Ksar Akil (Lebanon) and Implications for the Colonization of Europe by Anatomically Modern Humans
Source: PLoS One. 2013 Sep 11;8(9):e72931. doi: 10.1371/journal.pone.0072931 (PMC3770606; doi:10.1371/journal.pone.0072931)

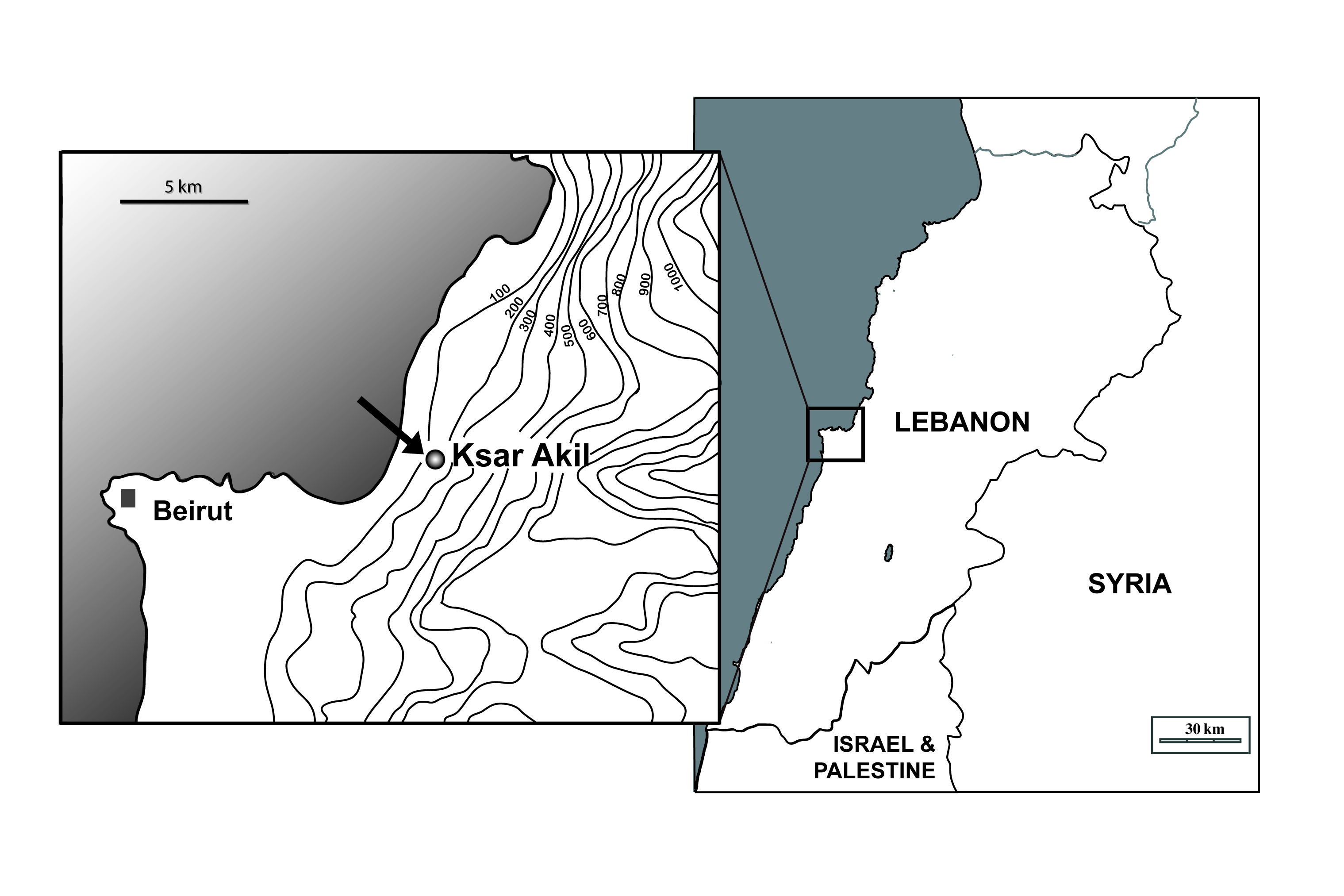

Supplement: Figure S1 — Location of the Ksar Akil site, Lebanon, a few kms NE of Beirut. (TIF) [file pone.0072931.s001.tif]

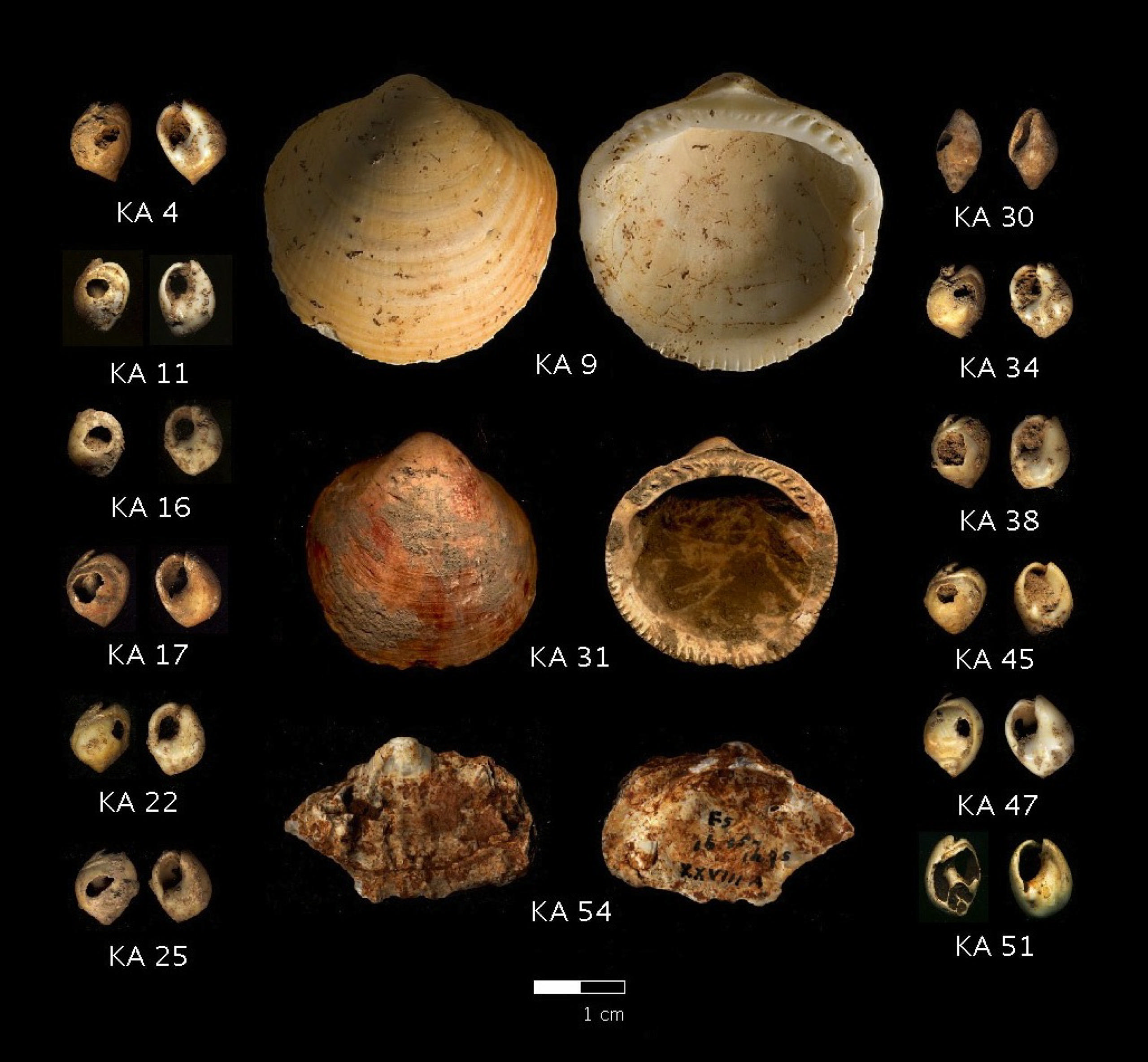

Supplement: Figure S2 — Examples of the dated shell specimens from Ksar Akil. The vast majority consists of beads of Nassarius gibbosulus/ circumcinctus while KA 30 is an example of Columbella rustica shell. KA 54, an Ostrea sp. shell, is one of the very few shells coming from Middle Palaeolithic layers. (TIF) [file pone.0072931.s002.tif]

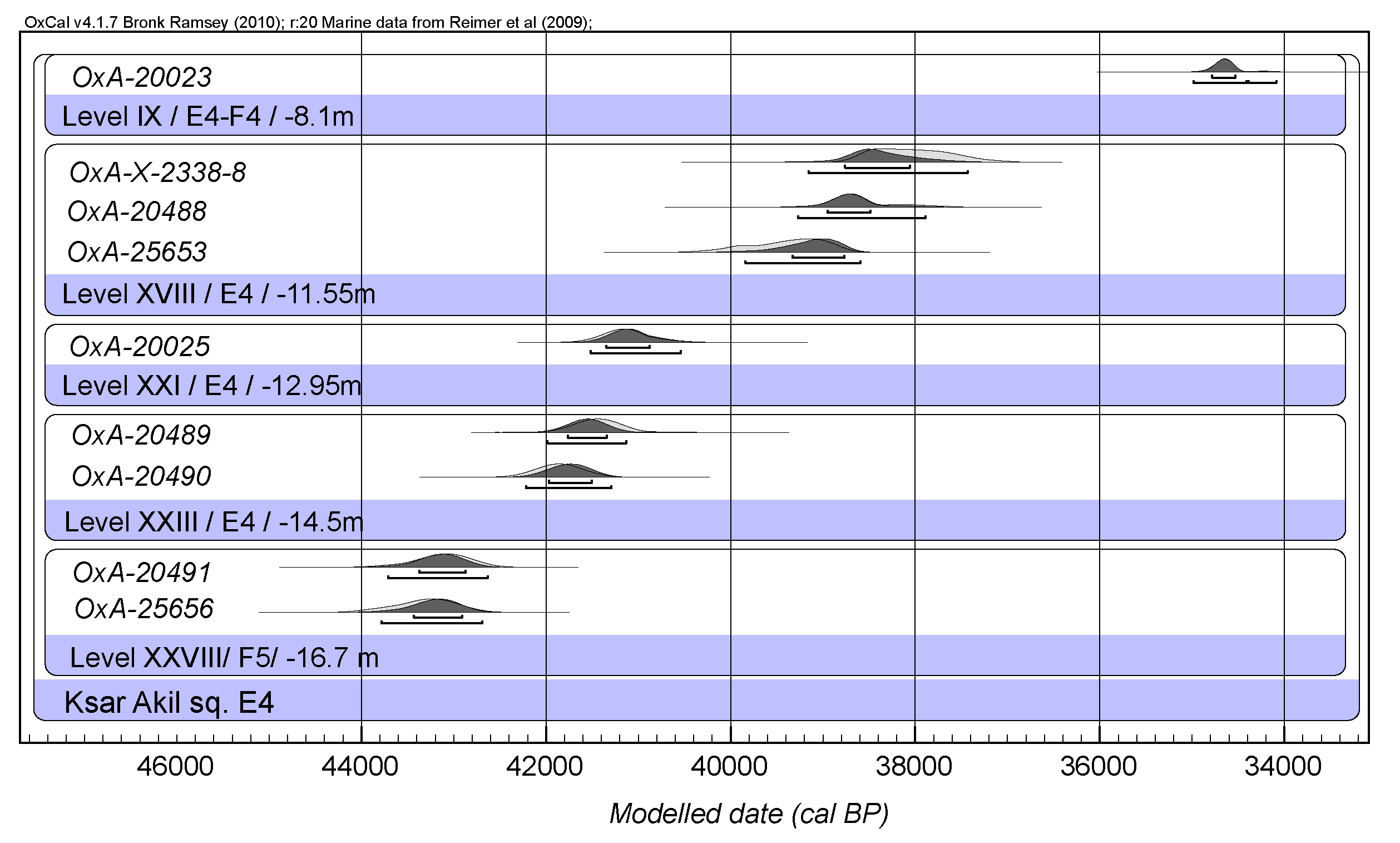

Supplement: Figure S3 — Plot of all available dates from square E4. OxA-20491 and OxA-25656 come from adjacent square (F5) and are used here as a terminus post quem. The determinations are plotted here together in order to check the chronological variation among specimens deriving from the same excavation square. The dates are consistent with the stratigraphic position. Assuming constant sedimentation (an over-simplified scenario), we may calculate an accumulation rate of 0.88 m of sediment deposited at the site every 1000 years. (TIF) [file pone.0072931.s003.tif]

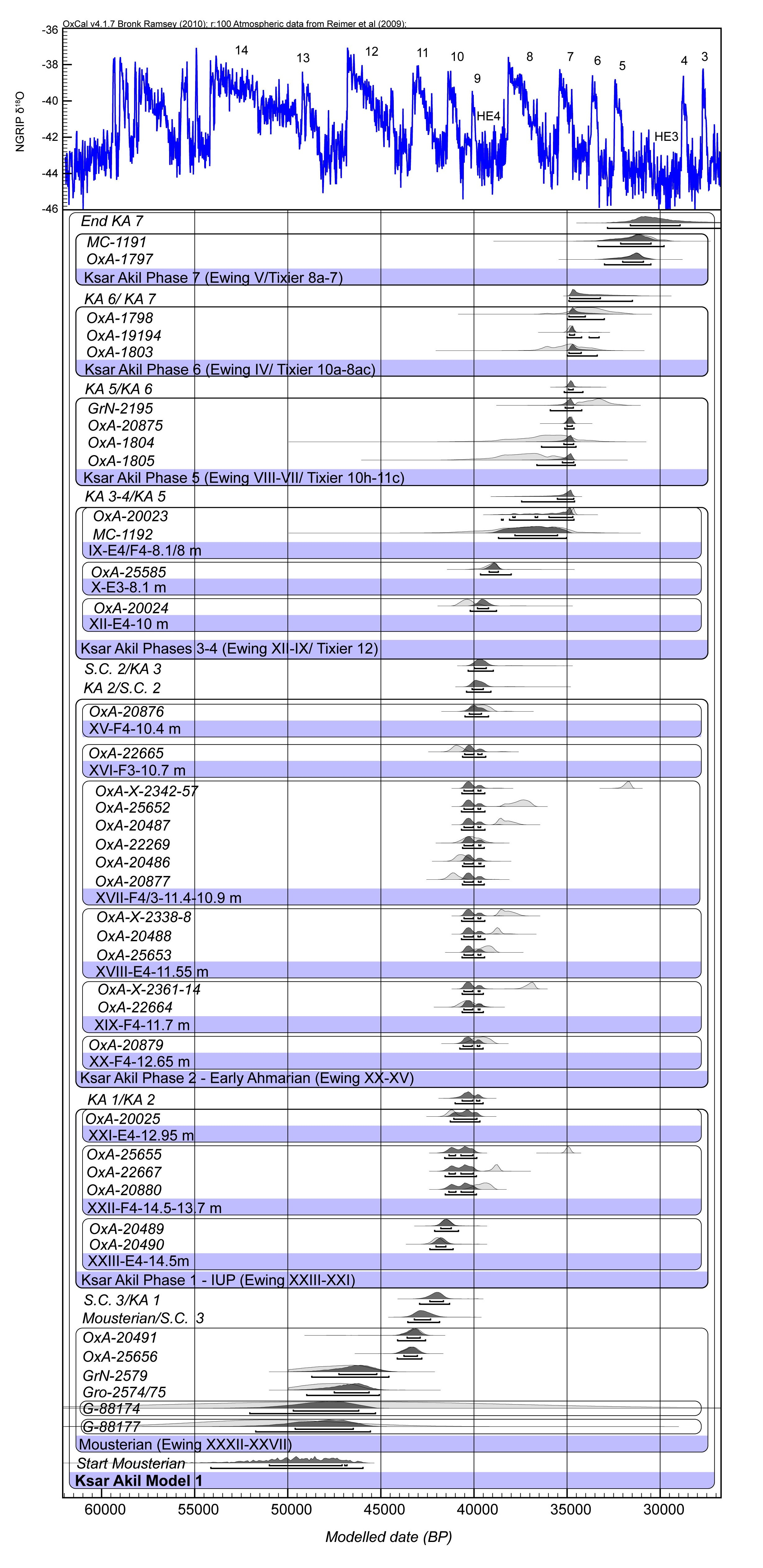

Supplement: Figure S4 — Bayesian Model 1. Initial Bayesian plot with all new dates, as well as previously obtained ones from the Tixier excavations. The model is structured around individual layers and phases. Of the 39 determinations, 11 are flagged as outliers. (TIFF) [file pone.0072931.s004.tiff]

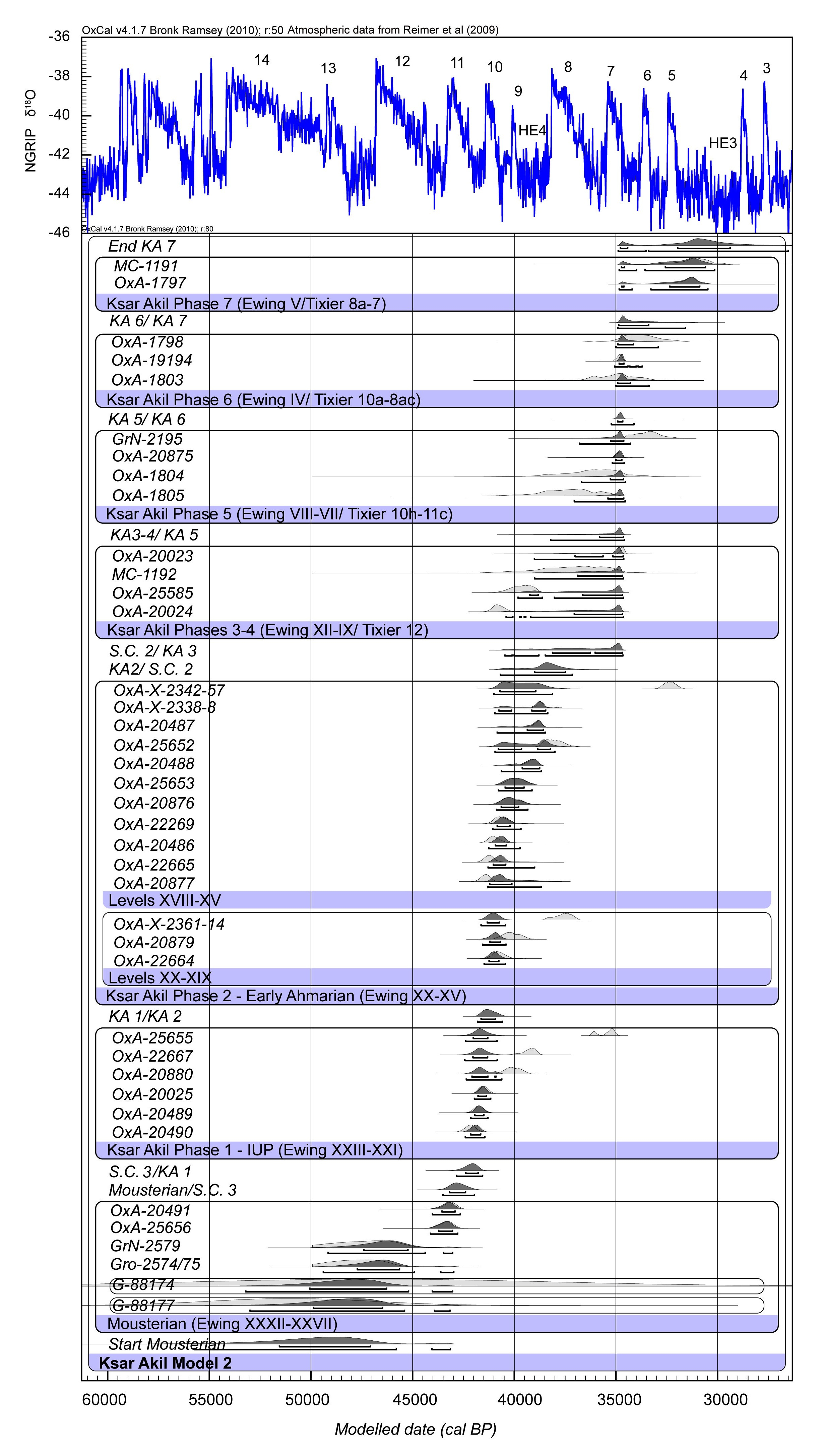

Supplement: Figure S5 — Bayesian Model 2. Second modeling iteration containing most available dates from the Early Upper and Middle Palaeolithic levels of the site, including previously obtained dates. Here, individual layers are grouped together within broad industrial phases (see text for details). Of the 39 determinations, 9 outliers are identified. (TIFF) [file pone.0072931.s005.tiff]
