# Supplementary material for: Chronology of Ksar Akil (Lebanon) and Implications for the Colonization of Europe by Anatomically Modern Humans
Source: PLoS One. 2013 Sep 11;8(9):e72931. doi: 10.1371/journal.pone.0072931 (PMC3770606; doi:10.1371/journal.pone.0072931)
Supplement: Table S1 — Archeological correlation of the Ksar Akil sequence, Boston College excavations (1937–1938, 1947–1948) and Tixier excavations (1969–1975). (DOC) [file pone.0072931.s006.doc]

| **BOSTON COLLEGE EXCAVATIONS** | | | | **TIXIER EXCAVATIONS** | | |
| --- | --- | --- | --- | --- | --- | --- |
| **Ksar Akil Phases (Boston College Excavations)** | **1937-1938 Field Season Levels** | **1947-1948 Field Season Levels** | **Assemblage Characteristics** | **Ksar Akil Phases (Tixier Excavations)** | **1969-1975 Excavation Levels** | ***Outils Caracteristiques*** |
| **7** | Not studied | VIb, VIa | Multiple reduction strategies including typical blade/bladelet manufacture and twisted bladelet manufacture from lateral carinated pieces; numerous retouched bladelets; burins and scrapers present in nearly equal numbers. | **III** | *Couche* 7 à 8a | Numerous retouched bladelets, scrapers and burins; burins typically flat-faced and multifaceted, and burins on truncation are larger; end-scrapers on unretouched blades |
| **6** | VI | IXa, VIIIc, VIIIb, VIIIa, VIIh, VIIg, VIIf, VIIe, VIId, VIIc, VIIb, VIIa | Multiple reduction strategies, twisted bladelet manufacture with lateral carination and twisted bladelet cores, elevated burin index, burins on concave truncation, Dufour bladelets | **IV** | *Couche* 8ac-10a | Truncation burins on small, thick flakes |
| **Not identified** | Not identified | IXb, IXa, VIIIc, VIIIb, VIIIa | Multiple reduction strategies, twisted bladelet manufacture with lateral carination and twisted bladelet cores, elevated burin index, burins on concave truncation, Dufour bladelets | **V** | *Couche* 10b-10g | Flat-faced multifaceted burins (*i.e.* Laterally carinated pieces) and sharp rise in bladelets with semi-abrupt, alternate retouch (*i.e.* Dufour bladelets) |
| **5** | VIII and VII | Xb, Xa, IXf, IXe, IXd, IXc | Multiple reduction strategies, flake production, predominantly blades and bladelets with straight or curved profiles, but twisted débitage also frequent, elevated scraper index, nosed and shouldered scrapers, tiny retouched bladelets, bone and antler tools | **VI** | *Couche* 10h-11bc | Nosed, shouldered and carinated scrapers, various side-scraper types, decline in retouched bladelets, although still abundant |
| **4** | X and IX | XIb, XIa, Xc | Multiple reduction strategies, blades and bladelets with straight and curved profiles, reduction in twisted débitage, elevated scraper index, retouched bladelets, el-Wad points and variants | **VII** | *Couche* 12 | El-Wad points, simple end-scrapers, nosed and shouldered scrapers, rare Aurignacian blades, and few burins |
| **3** | XIII-XI | XII | Multiple reduction strategies, twisted blades and bladelets, elevated burin index, flat-faced carinated burins, lateral carinated scrapers and burins, twisted el-Wad points |  | Not excavated |  |
