# Supplementary material for: Chronology of Ksar Akil (Lebanon) and Implications for the Colonization of Europe by Anatomically Modern Humans
Source: PLoS One. 2013 Sep 11;8(9):e72931. doi: 10.1371/journal.pone.0072931 (PMC3770606; doi:10.1371/journal.pone.0072931)
Supplement: Table S2 — Previous chronometric determinations from Ksar Akil. The majority of the dates relate to the late Upper Palaeolithic layers and were obtained on material from Tixier's excavation. (DOC) [file pone.0072931.s007.doc]

**Table S2 Previous determinations from Ksar Akil.** The majority of the dates relate to the late Upper Palaeolithic layers and were obtained on material from Tixier’s excavation.

| Lab. code | ^14^C | ± | Level/ depth | Material | Excavator |
| --- | --- | --- | --- | --- | --- |
| OxA-1791 | 23170 | 400 | 3 upper | Charcoal | Tixier |
| OxA-1792 | 22850 | 400 | 3b major | Charcoal | Tixier |
| OxA-1793 | 22050 | 360 | 3b lower | Charcoal | Tixier |
| OxA-1794 | 22480 | 380 | 3bb | Charcoal | Tixier |
| OxA-1795 | 22850 | 380 | 3c | Charcoal | Tixier |
| OxA-1796 | 21100 | 500 | 7bb | Charcoal | Tixier |
| OxA-1797 | 26900 | 600 | 8a | Charcoal | Tixier |
| OxA-1798 | 29300 | 800 | 8ac | Charcoal | Tixier |
| OxA-1803 | 30250 | 850 | 9a | Charcoal | Tixier |
| OxA-1804 | 31200 | 1300 | 10 lower | Charcoal | Tixier |
| OxA-1805 | 32400 | 1100 | 11bm | Charcoal | Tixier |
| MC-1191 | 26500 | 900 | 8ai | Charcoal | Tixier |
| MC-1192 | 32000 | 1500 | 12.0 m | Charcoal | Tixier |
| MC-410 | 24400 | 900 | 3b/ 2.80-2.90 m | Landsnail | Tixier |
| MC-411 | 14100 | 500 | C/ 3.50-3.55 m | Bone | Tixier |
| MC-? | 28600 | 680 | Phase V | n/a | Tixier |
| MC-686-88 | 27000 |  | Phase V | n/a | Tixier |
| MC-679 | 27350 |  | Phase VI | Charcoal | Tixier |
| MC-574-580 | ? | ? | Dates not published | Shell? | Tixier |
| GrN-2195 | 28840 | 380 | 6-7.5 m | Shells | Ewing, 1948 open section |
| GrN-2579 | 43750 | 1500 | XXVI or XXVII/ 16 m | Clay treated as charred matter | Ewing, 1948 open section |
| Gro-2574/75 | 44400 | 1200 | XXVII/ 16 m Red clay beneath Stone Complex 3 | Clay | Ewing, 1948 open section. Same as above ? |
| G-88174 | 47000 | 9000 | XXVI BE V | U-series/ bone surface | 1947-8? |
| G-88173 | 19000 | 5000 | XXVI BE V | U-series/ bone bulk | 1947-8? |
| G-88177 | 51000 | 4000 | XXXII FV | U-series/ bone surface | 1947-8? |
| G-88178 | 49000 | 5000 | XXXII FV | U-series/ bone bulk | 1947-8? |
