# Supplementary material for: Chronology of Ksar Akil (Lebanon) and Implications for the Colonization of Europe by Anatomically Modern Humans
Source: PLoS One. 2013 Sep 11;8(9):e72931. doi: 10.1371/journal.pone.0072931 (PMC3770606; doi:10.1371/journal.pone.0072931)
Supplement: Table S3 — New radiocarbon determinations from Ksar Akil and details for stratigraphic details for each sample. KA 51 was dated twice as it underwent mineralogical separation (see [32]) due to the presence of calcite in the original fraction. In the last column the percentage of secondary calcite in the shell matrix, established by XRD analysis, is indicated. The differentiation between Nassarius gibbosulus or Nassarius circumcinctus was not always possible due to the preservation state of the shells; here they are all tentatively ascribed to the former species. The δ13C value is also given when this was unusual for marine shells, therefore indicating either some degree of meteoric diagenesis or other technical issues. The 3 determinations marked with an asterisk were not used in the modeling since they are most certainly problematic (see text). (DOC) [file pone.0072931.s008.doc]

**Table S3 New radiocarbon determinations from Ksar Akil and details for stratigraphic details for each sample. KA 51 was dated twice as it underwent mineralogical separation (see reference 32) due to the presence of calcite in the original fraction. In the last column the percentage of secondary calcite in the shell matrix, established by XRD analysis, is indicated. The differentiation between *Nassarius gibbosulus* or *Nassarius circumcinctus* was not always possible due to the preservation state of the shells; here they are all tentatively ascribed to the former species. The δ^13^C value is also given when this was unusual for marine shells, therefore indicating either some degree of meteoric diagenesis or other technical issues. The 3 determinations marked with an asterisk were not used in the modeling since they are most certainly problematic (see text).**

| **Sample** | OxA | **^14^C date** | **±** | **Level, Sq., Depth in m** | **Genus/ Species [δ^13^C** **‰]** | **Calibrated (95.4%)**  **from to** | | **Aragonite-**  **Calcite%** |
| --- | --- | --- | --- | --- | --- | --- | --- | --- |
|  |  |  |  |  |  |  | |  |
| **Charc.** | 19194 | 30250 | 170 | 8ac=VI | Not identified | 35110 | 34580 | n/a |
| **KA 4** | 20875 | 30640 | 160 | VIII, G 3-4, -6.75 | *Nassarius gibbosulus* | 35090 | 34550 | 100-0 |
| **KA 9 *** | 20022 | 37210 | 230 | IX, G 4, -8 | *Glycymeris* sp. | 42150 | 41270 | 100-0 |
| **KA 11** | 20023 | 30360 | 140 | IX, E 4-F 4, -8.1 | *Nassarius gibbosulus* | 34960 | 34070 | 99.9-0.1 |
| **KA 15** | 25585 | 34550 | 250 | X, F 3, -8.1 | *Nassarius gibbosulus* | 40050 | 38480 | n/a |
| **KA 16** | 20024 | 35520 | 200 | XII, E 4, -10 | *Nassarius gibbosulus* | 40940 | 39350 | 99.9-0.1 |
| **KA 17** | 20876 | 35020 | 240 | XV, F 4, -10.4 | *Nassarius gibbosulus* | 40400 | 38820 | 100-0 |
| **KA 18** | 22665 | 36040 | 240 | XVI, F 3, -10.7 | *Nassarius gibbosulus* | 41410 | 40170 | 100-0 |
| **KA 30** | X-2342-57 | 28130 | 110 | XVII, F 3, -10.9 | *Columbella rustica* **[-5.1]** | 32270 | 31380 | 99.9-0.1 |
| **KA 31** | 20877 | 36270 | 240 | XVII, F 3, -10.9 | *Glycymeris* sp. | 41560 | 40410 | 99.7-0.3 |
| **KA 27** | 22269 | 35390 | 250 | XVII, F 3, -10.9 | *Acanthocardia* sp. | 40860 | 39100 | 99.8-0.1 |
| **KA 25** | 20487 | 33930 | 220 | XVII, F 3, -10.9 | *Nassarius gibbosulus* | 38880 | 37380 | 99.5-0.5 |
| **KA 29** | 25652 | 33300 | 230 | XVII, F 4, -11.25 | *Columbella rustica* | 38760 | 37200 | 100-0 |
| **KA 22** | 20486 | 35780 | 240 | XVII, F 4, -11.25 | *Nassarius gibbosulus* | 41200 | 39610 | 100-0 |
| **KA 37** | X-2338-8 | 33760 | 210 | XVIII, E 4, -11.55 | *Columbella rustica* | 38760 | 37200 | 99.8-0.2 |
| **KA 34** | 25653 | 34830 | 240 | XVIII, E 4, -11.55 | *Nassarius gibbosulus* | 40220 | 38700 | 99.8-0.2 |
| **KA 35** | 20488 | 34230 | 210 | XVIII, E 4, -11.55 | *Nassarius gibbosulus* | 39250 | 37660 | 100-0 |
| **KA 38** | 22664 | 35510 | 240 | XIX, F 4, -11.7 | *Nassarius gibbosulus* | 40980 | 39280 | 100-0 |
| **KA 39** | X-2361-14 | 32960 | 160 | XIX, F 4, -11.7 | *Columbella rustica* | 37580 | 36530 | 99.7-0.3 |
| **KA 41** | 20879 | 35010 | 240 | XX, F 4, -12.65 | *Nassarius gibbosulus* | 40390 | 38810 | 100-0 |
| **KA 45** | 20025 | 36390 | 210 | XXI, E 4, -12.95 | *Nassarius gibbosulus* | 41630 | 40560 | 100-0 |
| **KA 49** | 25655 | 30890 | 160 | XXII, F 4, -13.7 | *Columbella rustica* | 36270 | 34890 | 99.5-0.5 |
| **KA 47** | 20880 | 34940 | 200 | XXII, F 4, -13.7 | *Nassarius gibbosulus* | 40260 | 38790 | 100-0 |
| **KA 48** | 22667 | 34320 | 190 | XXII, F 4, -13.7 | *Nassarius gibbosulus* | 39410 | 37910 | 99.9-0.1 |
| **KA 51** | 20489 | 36790 | 270 | XXIII, E 4, -14.5 | *Nassarius gibbosulus* | 41960 | 40950 | 20-80 |
| **KA 51** | 20490 | 37430 | 320 | XXIII, E 4, -14.5 | *Nassarius gibbosulus* | 42380 | 41320 | 93-7 |
| ***KA 54 **** | *X-2361-17* | *33810* | *180* | XXVIII, F 5, -16.55–75 | *Ostrea* sp. **[-3.4]** | 38770 | 37330 | 0-100 |
| ***KA 54 **** | *X-2344-23* | *35900* | *400* | XXVIII, F 5, 16.55–75 | *Ostrea* sp. **[-2.6]** | 41480 | 39440 | 0-100 |
| **KA 54** | 20491 | 39310 | 330 | XXVIII, F 5, 16.55–75 | *Ostrea* sp. **[1.6]** | 43830 | 42520 | 0-100 |
| **KA 55** | 25656 | 39530 | 330 | XXVIIIA, F 5, 16.55–75 | *Ostrea* sp. **[1.0]** | 44280 | 43020 | n/a |
