# Supplementary material for: Chronology of Ksar Akil (Lebanon) and Implications for the Colonization of Europe by Anatomically Modern Humans
Source: PLoS One. 2013 Sep 11;8(9):e72931. doi: 10.1371/journal.pone.0072931 (PMC3770606; doi:10.1371/journal.pone.0072931)
Supplement: Text S1. — (DOC) [file pone.0072931.s009.doc]

**SUPPORTING INFORMATION**

**Section I: The site of Ksar Akil and further details of the archaeological sequence**

**Section II: Radiocarbon dating**

**Section III: Bayesian-based chronological framework**

**References**

**Section I:**

**Ksar Akil: The site and archaeological sequence**

***a. Location and history of research***

The Ksar Akil rockshelter (other spellings include: Ksar 'Akil, Ksâr 'Akil, Ksar 'Aqil) is located 10 km northeast of Beirut (Fig. S1), just above the coastal plain in the foothills of the Lebanon Mountain range. The site is situated at the base of a high limestone cliff on the north bank of the Antelias River valley.

The investigations at Ksar Akil have now spanned nearly 90 years, beginning somewhat inauspiciously when the owner of the land probed the site for treasure in 1922 and dug through 15 meters of deposits. It was not until 1937 that the first large-scale and scientific excavations began on the advice of the Abbé Henri Breuil [1]. The archaeological team came from Boston College, Massachusetts and was directed by Joseph G. Doherty, S.J. [2, 3]. Doherty, a student of Dorothy Garrod at Cambridge University, was assisted by J. Franklin Ewing, S.J., of Fordham University who joined the team as paleontologist and anthropologist in June 1938 [2, 3]. Ewing assumed the directorship of the project for the 1947-1948 field seasons, suggesting these early investigations by the Jesuit priests actually represent the efforts of both Boston College and Fordham University.

An extensive series of photographs illustrating the 1937-1938 excavations was published by Bergman [4, 5], using images supplied to Garrod by Doherty [6]. Some of these have now become publicly available through the Pitt Rivers Museum, University of Oxford, after the recent digitization of D. Garrod’s photographic archive (http://web.prm.ox.ac.uk/garrod/). Based on the surviving documentation, it is apparent that the field methodology does not measure up to modern standards, although for the time it was relatively rigorous.

The excavation grid was mapped out in 16 two-meter squares, with alphabetic letters designating the east-west axis, and numbers designating the north-south axis [3, 5]. Shovel skimming was the primary means of excavation [5] along with troweling of wall sections, while delicate objects were examined with brush and trowel [5]. Sediments were sieved through a medium mesh and the artefacts boxed or bagged by provenience unit [5]. Since most specimens were heavily encrusted with breccia, each artefact was brushed, scraped and washed [1]. It is estimated that perhaps as many as 2,000,000 lithic artifacts, bone and antler tools, elements of personal ornamentation, such as shell beads, as well as faunal remains were recovered in the two field seasons.

Doherty, guided by the profile visible in the 1920s-era treasure-hunter’s pit, used geological layers to define the stratigraphy. These were often of substantial size, often with a thickness of between 1 and 2 meters, and did not necessarily align with the archaeological levels [7, 8]. These excavations reached 23 meters below datum, and revealed 36 levels, I-XXXVI, from top to bottom (Fig. 1, Main Text).

In the subsequent 1947-1948 field seasons, Ewing developed a more refined stratigraphy and concentrated on collecting a greater variety of artifacts, specifically the miniscule bladelets common in the upper portions of the Ksar Akil sequence [8, 9]. For the later Upper Palaeolithic levels, this resulted in Doherty’s original stratigraphy involving only eight levels (XIII-VI) being subdivided into 27 levels [8].

In 1969, Tixier began a series of investigations at Ksar Akil [7, 10, 11]. Using careful excavation techniques, such as the three-dimensional recording of artifacts, he established a finely divided stratigraphy and recognized a series of *sols d’habitat* near the shelter wall. During the course of work that ended prematurely in 1975, Tixier reached a depth of nearly 9 meters and identified 30 different levels. His vastly refined stratigraphy is evidenced by the fact that he identified 12 discrete levels within the equivalent elevations of Level VIII from the 1937-1938 investigations. Based upon the published sections from the Boston College and the 1969-1975 excavations, it would appear that the deepest level excavated by Tixier between 1969 and 1975 is equivalent to levels X-IX of the 1937-1938 investigations, or levels XI-Xc of the 1947-1948 season [5, 8].

Overall it is estimated that about 700 m^3^ of deposit have been removed from the site. According to the geological observations of Wright [12, 13], there is strong evidence that climatic variability affects soil formation and occupation patterns. The three “Stone Complexes” may be indicative of this.

The actual processes responsible for the formation of the Stone Complexes have been seen as the result of the effect of “pluvial glaciation” on the weakening of limestone surfaces [12]. Ewing himself and other subsequent researchers supported that the intercalated red clay deposit represents disintegrated and decalcified limestone that separates stony levels above and below, the latter coming from the overhanging roof of the shelter and therefore very likely being the result of varying conditions of humidity (and seasonal variations of temperature) [12]. Azoury [1], on the other hand, suggests that since there is alluvial sand and pebbles in the deposits below a depth of 16 m (in the MP levels just below Stone Complex 3), the red clay deposit may have been introduced by the stream rather than having been developed in situ by soil weathering process. In our opinion, both explanations remain tentative and need to be substantiated by further in situ observations and modern analytical techniques.

***b. Archaeological Sequence***

*Middle Palaeolithic :1937-1938 Season, XXXVI-XXVI*

The first Middle Palaeolithic occupation, from level XXXVI to level XXVI (19.4–15 m below datum, respectively), took place while the rockshelter was intermittently flooded by the nearby stream [12]. The alluvial deposits stop between -17 m and -16 m when the course of the stream changed and habitation of the rockshelter became more stable and dense [14].

Copeland [5] assigned the assemblage to Phase 3/ Layer B (and possibly Phase 2/ Layer C) Tabun Mousterian, while Marks and Volkman [15] assigned the lower levels (XXVIIIB and XXVIIIA) to Phase 1/ Tabun D Mousterian based on the presence of ovoid blanks, Levallois and discoid cores, and Mousterian tool types, while the uppermost levels (XXVIIB to XXVIA) were assigned to Phase 2/ Layer C type Mousterian due to the presence of blade and point forms.

*Initial Upper Palaeolithic: 1937-1938 Season, XXV-XXI / Ksar Akil Phase 1*

The earliest Upper Palaeolithic occupations at Ksar Akil, levels XXV-XXI, have been regarded as part of the transition from the Middle to Upper Palaeolithic [16, 17]. It should be noted that many authors have used the term ”Transitional” to describe similar industries, while recognizing the Upper Palaeolithic character of both the technological operating chains and tool typology [18, 19]. We agree with recent characterization of these assemblage types as Initial Upper Palaeolithic (IUP) by scholars [20]. A possible occupational hiatus in levels XXV-XXIV, identified as “Stone Complex 3”, possibly indicates abandonment of the site and separates the latest Mousterian and first Initial Upper Palaeolithic levels.

There seem to be two distinct stages represented in the Initial Upper Palaeolithic layers, which collectively are referred to as Upper Palaeolithic Phase I by Ohnuma and Bergman [17]. The sample from levels XXV-XXIV is small, but characterized by opposed platform cores with parallel sides. Levels XXIII-XXI, on the other hand, contained numerous single platform blade cores with faceted platforms and converging sides. The triangular shape of the cores cause blade removals to converge, resulting in the production of blanks morphologically similar to elongated Levallois points. The blow used to detach the blades in these levels is well on to the striking platform (non-marginal flaking), resulting in relatively thick blanks with large butts. An analysis of the ventral surfaces of the blanks in these levels suggests that most blades were detached with soft hammers [17].

The assemblages in levels XXV-XXI are almost entirely composed of Upper Palaeolithic tool types including varying percentages of chamfered pieces. This artifact type, generally rare in the rest of the Levant, is made by a tranchet blow at the proximal or distal end of a flake or blade [21]. Other components of the tool kits include end-scrapers and truncation burins, which are always more numerous than the dihedral types [6]. Outside of the Lebanese sites situated along the coastal plain, such as Abu Halka in northern Lebanon, good comparative material for the Initial Upper Palaeolithic at Ksar Akil has been found at Üçagizli cave, southern Turkey [19, 22].

*Early Ahmarian: 1937-1938 Season, XX-XVI / Ksar Akil Phase 2*

Although no clear-cut technological distinction exists between level XX and those immediately preceding it, there is a shift from cores with single, faceted platforms and converging sides to parallel-sided cores with opposed, plain platforms [17]. Cresting and the core tablet technique begin to be used more often for preparation and maintenance. The blade blanks tend to be much thinner than in levels XXV-XXI and are produced by striking quite close (marginal flaking) to the edge of the core platform, resulting in tiny butts. In order to avoid damaging the platform, abrasion is used extensively to remove overhang and thicken and strengthen the edge. The blades in these levels are believed to have been detached with soft hammers, probably by direct percussion.

Levels XX-XVI, Ksar Akil Phase 2, have tool assemblages consisting of end-scrapers and retouched blades and bladelets including backed and partially backed blades, as well as robust el-Wad points (over 15% of the tool kits) and *pointes à face plane*. The *pointe à face plane* at Ksar Akil [4, 6] is a leaf-shaped piece formed by invasive retouch, which has also been recognized in a remarkably similar Early Ahmarian assemblage at Üçagizli Cave by Kuhn et al. [19, 22]. A curious feature of all these levels is the relative scarcity of burins; the burin index for levels XVIII-XVI ranges between 1 and 1.5 [6]. It is widely agreed that Phase 2 fits the description of the Leptolithic lineage, Ahmarian industry, and has recently been described as its northern facies [8, 17, 18, 23].

*Stratigraphic Hiatus: 1937-1938 Season, XV and XIV*

Above level XVI is level XV, part of “Stone Complex 2,” while the following level XIV represents a major break in the occupation of the site [6]. Both were quasi-sterile. Herbert Wright [13] indicated that at about 10.5 meters below datum there was a “*distinct layer of red clay about 30 cm thick overlain by a layer of angular stones of equal thickness. The red clay has the appearance of the typical terra rosa developed on the limestone in the Jurassic bedrock…* [and] *is considered to be a soil developed in situ in the rock shelter*.” The exact palaeoclimatic significance of Stone Complex 2 was difficult for Wright to interpret, but he felt that it may represent an episode of intensive weathering, such as during a pluvial phase, coinciding with a period when the rockshelter was not occupied allowing for development of the soil. Regardless of the mechanisms of formation, Stone Complex 2 coincides with a break in the cultural sequence that separates the Early Ahmarian industries of levels XX-XVI from the later Upper Palaeolithic levels, XIII-VI [4].

*Later Upper Palaeolithic :1937-1938 Season, XIII-VI; 1947-1498 Season, XII –VI a and b / Ksar Akil Phases 3-6*

The upper portion of the Ksar Akil sequence, comprising levels XIII-VI of the 1937-1938 field seasons and levels XII-VIa and b of the 1947-1948 field season, spans approximately 7.25 meters of deposits from 10.65 meters to about 3.40 meters below datum. Currently the descriptor “Aurignacian” has been removed from these layers and researchers (e.g. [8]) prefer the phase designations as summarised in Table S1.

This upper portion of the sequence is stratigraphically and culturally complex (Table S1). While the Early Ahmarians at Ksar Akil successfully produced the full range of blanks required for tools within the framework of blade core reduction (e.g., flakes, core tablets, blades and bladelets), this is seemingly not the case for later Upper Palaeolithic people at the site. Specifically, each of the levels incorporated into Phases 3-6 is characterized by multiple operating chains for the production of blades and/or bladelets with twisted profiles, as well as straight or curved profiles. These involve objects clearly intended for use as cores, as well as multifaceted burins and a variety of scrapers such as the carinated and thick nosed and shouldered types, all of which occur in varying frequencies in levels XIII-VI. As suggested by the work of Chiotti [24] and Hays and Lucas [25], carinated and multifaceted burins and nosed and shouldered scrapers appear to be multipurpose, serving as both cores and tools.

There have been considerable problems aligning the upper part of the Ksar Akil sequence with the rest of the Levant with the notable exception of levels VIII and VII (Phase 5) and level VI (Phase 6). These are widely considered to represent the Levantine Aurignacian, *sensu stricto*, and the Atlitian, respectively. Whether the blade and bladelet dominated industries of Phase 3 (Levels XIII-XI) and Phase 4 (Levels X and IX) represent developmentally earlier stages of the Aurignacian, as posited by Tixier and Inizan [7, 26], still remains a matter of debate.

**Section II:**

**Radiocarbon dating**

***a. Previous chronology for Ksar Akil***

Prior to this work there were 26 radiometric dates from the site, from the later Upper Palaeolithic and the Middle Palaeolithic layers but none from the IUP or Early Ahmarian ones (SI Table S2). These dates were produced in three radiocarbon facilities (Oxford, Groningen and Monaco) and the vast majority relate to Tixier’s excavations [26]. No radiocarbon dates existed for material from the older excavations although two radiocarbon determinations (GrN-2579 and GrN-2195) were made on clay and shell samples, respectively, collected in 1959 from Ewing’s 1948 open sections [27]. A date (“Gro-2574/75”) is mentioned by Ewing but no other record exists for it; it is very likely that this is the same date as GrN-2579. Finally, four U-series dates on two Mousterian bones from the bottom of the sequence were produced by van der Plicht et al. [28], but the results were inconclusive.

During the late 1980s, a series of bones from Ewing’s excavations submitted to Oxford for AMS radiocarbon dating but failed to produce any collagen. Similarly, in 2008, we sampled 10 bone points recovered by Tixier and curated at the University of Bordeaux I. We analysed the points for their N content and calculated the C:N atomic ratios, both used as a proxy for collagen presence and state of preservation. Again no collagen was identified in any of the sampled implements and the analytical values ranged much beyond accepted levels (%N=0.2-0.21 and C:N=20-91; where accepted values are %N=>0.75-1 and C:N=<~7; see [29]).

No charcoal was collected at the time of excavation despite Ewing explicitly mentioning the presence of structured hearths, both in the Middle and Upper Palaeolithic levels.

The abundance of marine shells throughout the stratigraphy (with the exception of the lower Middle Palaeolithic layers, XXXVII–XXIX) and recent advances in the reliable dating of this type of material [30] gave rise to the present work.

***b. New radiocarbon chronology***

***Materials for dating***

The Upper Palaeolithic molluscan collection of Ksar Akil contains impressive numbers of both marine and terrestrial landsnails. The shells from the early excavations, about 2000 specimens, were studied initially by Altena Van Regteren [31] at the Rijksmuseum van Natuurlijke Historie in the Netherlands, where the material was sent by Ewing himself. Inizan and Gaillard [32] studied about 200 shells from Tixier’s recent excavations, from the upper stratigraphic levels. Forty-five molluscan species were identified; *Nassarius gibbosulus, Columbella rustica*, *Osillinus turbinatus* and *Glycymeris* sp. are the most abundant throughout the sequence. Ewing [33] mentions thousands of shells of the terrestrial snail *Helix* sp., as well as marine shells of *Patella* sp. and *Trochus* sp., from –13 m. (~XIX) upwards. These are not accounted for in the aforementioned studies nor are currently present in such numbers in the surviving collection. It is possible that only a portion of the invertebrate remains made their way out of Lebanon. An interesting comparison of the molluscan assemblage from Ksar Akil with that of Üçağızlı, in the northern Levant, was reported by Kuhn et al. [34].

In 2007, the material studied by Altena Van Regteren (31) and “lost” since, was located by us at the Naturalis Natural History Museum (Leiden, The Netherlands). The shells appear to derive from both 1937-38 and 1947-48 excavations, since, according to Hooijer [35], material from the former campaign is only accompanied by depth (3-15.4 m) and square information (E5, F3, F5), while material from the latter season has been ascribed level information too.

We favoured samples with the best possible recording and with the best preservation state. We selected 54 shells from levels V–XVIII, of which 26 were dated (Fig. S2). Most of these are beads on gastropods, either *Nassarius gibbosulus* or *Columbella rustica*, but a few bivalves (*Glycymeris* and *Acanthocardia* sp.) were also dated to check inter-species age differences and to elucidate modes of exploitation. Many shells preserved original pigmentation, while some had ochre residues on them (e.g. KA 31 and KA 51, Fig. S2). A *Glycymeris bimaculata* (KA 9) bore traces of deliberate edge modification, possibly for use as a scraper [36].

The pretreatment methods used to date the samples in Oxford were briefly described in the main text, as well as in recent publications [30, 37].

***Results***

Overall, 30 new radiocarbon dates were produced: 29 on shell (KA 51 was dated twice and KA 54 thrice) and one on charcoal. The new determinations are shown in Table S3 along with the calibrated ranges obtained using the latest calibration curve, IntCal09 for the terrestrial charcoal sample and IntCal09-Marine for the shells [38]. A constant marine reservoir of 400 ^14^C years is included in the marine curve and, in addition, we corrected the shell measurements for the Mediterranean local reservoir (ΔR=58 ± 85 ^14^C years; [39]).

The lowermost stratigraphic unit for which four dates were obtained, is the Mousterian level XXVIII, at 16.6 m below datum, just below Stone Complex 3. An *Ostrea* sp. valve was dated three times due to problems identified with the carbonate mineral, and produced three different results (OxA-X-2344-23: 35900 ± 400; OxA-X-2361-17: 33810 ± 180; OxA-20491: 39310 ± 330 BP). The δ^13^C values for the first two, which are also the youngest, ranged from -2.6 to -3.8‰. This suggests some degree of meteoric diagenesis and incorporation of terrestrial carbon compounds. Unfortunately, shells of *Ostrea* sp. are predominantly calcitic in nature and XRD screening cannot help discriminate secondary, post-depositional mineral formation, such as low-Mg calcite. OxA-20491, the third date, was produced on a sample taken from a different part of the valve (away from surface layers sampled before and closer to the thicker umbo area) and this is the oldest determination. This is considered the most reliable of the three and its δ^13^C value falls within the expected range for marine carbonates (1.6‰). In order to determine securely the age of layer XXVIII, we obtained a second *Ostrea* sp. valve, from the same level (XXVIII). Based on morphometric comparisons this second valve could belong to the same animal as the previous specimen. The new date, OxA-25656: 39530 ± 330 BP, is identical to OxA-20491, and its δ^13^C value (1.0‰) is also comparable. Hence, we argue that the age of level XXVIII is around 39.5 ka BP.

This age estimate is younger than two previous dates produced in the 1960s at the Groningen Laboratory on clay treated as charred organic matter, also from 16 m below datum (Gro-2474/5 and GrN-2579, ~44 ka BP; Table S3). Sediment clay, however, is a problematic material for dating because it is impossible to determine with certainty where the measured carbon originates from. Additionally, conventional radiocarbon dates of this age produced fifty years ago, are very close to the background limits of the time. OxA-20491 and OxA-25656 may be considered a more realistic estimate for the age of the late Mousterian at the site. We should note here, however, that we cannot determine the antiquity of the earlier Mousterian levels, and if the U-series determinations from levels XXXII are to be trusted, then the Mousterian must have certainly started before 50 ka.

Seven dates were produced from the succeeding IUP (Phase 1). OxA-25655 (XXII) gave an unexpectedly young age (30890 ± 160 BP). This *C. rustica* shell was identified with some secondary calcite (about 0.5%) so the date may be an underestimate, although mixing is the most likely explanation, since this particular species has produced highly variable dates. The rest of the samples from Phase 1 range from 37.4 to 34.3 ka BP, the majority clustering around 35 ka BP. The two oldest determinations (OxA-20489 and OxA-20490) come from the same specimen (XXIII, 14.5 m) which was treated with the CarDS protocol [30]. They date to ~37 ka BP and are statistically identical. From overlying level XXII (13.7 m) come OxA-20880 and OxA-22667 that date to 34.9 and 34.3 ka BP, respectively, and they both overlap largely at 2σ (although marginally fail to pass the χ^2^ test). OxA-20025 from XXI (12.95 m) is older (~36 ka BP) but it is well within the range of dates for Phase 1.

According to Azoury [1) the passage from Phase 1 (IUP) to the earliest classic Upper Palaeolithic, Phase 2 (Early Ahmarian), is reached in level XX. This level was dated at ~35 ka BP (OxA-20879). Two dates were obtained from level XIX, OxA-22664 and OxA-X-2361-14, which are different from each other (35.5 and 33 ka BP respectively). The latter, made on a *C. rustica* shell, contained some secondary calcite. While it appears that layer XIX was associated with a rock fall episode [40] therefore post-depositional disturbances cannot be ruled out, we think that the young dates on *C. rustica* observed throughout the sequence may relate to post-excavation mixing.

The largest variation in results comes from levels XVIII and XVII. Most researchers consider these layers (as well as XVI) closely related; in the field they were difficult to separate or follow, which makes attribution of shell remains to specific archaeological contexts rather problematic. This is accentuated by the fact that the shell depth only refers to the start of the geological layer and not the actual depth the shells were found in.

Level XVIII is associated with three dates (OxA-X-2338-8, OxA-25653 and OxA-20488) ranging between 34.8 ka BP and 33.8 ka BP. Level XVII, on the other hand, gave slightly older ages, despite being stratigraphically higher. If we exclude one determination on *C. rustica* (diagenetic OxA-X-2342-57 at 28.1 ka BP) the dates for level XVII span from about 36 to 33.3 ka BP. It should be noted here that all dates from XVIII come from square E4 while the ones from XVII come either from F3 or F4. Since the levels were difficult to follow stratigraphically we ought to consider XVIII and XVII closely related. Level XVI is associated with a single date (OxA-22665) of 36 ka BP and level XV with OxA-20876 at 35 ka BP.

Ksar Akil Phase 3 starts in level XIII, directly on top of the stratigraphic break marked by Stone Complex 2. Two *Nassarius gibbosulus* shells, OxA-20024 from layer XII at 10 m below datum and OxA-25585 from layer X at 8.1 m below datum, were dated at 35.5 ka BP and 34.5 ka BP, respectively. If the Stone Complex represents a significant depositional hiatus (e.g., [12]) then these determinations appear to be too old. It is not possible to tell whether this is due to older material coming up the sequence from layer XV, ~ 60 cm below, whether the stratigraphy was difficult to follow or whether the shells were post-depositionally mixed or these are indeed ‘old’ shells. The latter is not supported by other evidence; for example, the specific *Nassarius* sp. shells (KA 15 and KA 16) do not show marks of beach-wear (pitting, polishing) or the natural abrasion which typifies aged shells. Two determinations from layer IX, (OxA-20022 and OxA-20023) yielded very different results (37.2 and 30.3 ka BP respectively). The first however was made on a *Glycymeris* valve, almost certainly collected old (semi-fossilized) from the beach. It is now known that *Glycymeris* valves found along the Levantine coast are frequently very old (see references [36, 41-42] for discussion) and may have never lived in the region but their shells were transported there by currents after death. Finally, a date from VIII also at 30.6 ka BP (OxA-20875) is identical to OxA-20023 from IX and we think that this is the correct age for levels IX-VIII.

It is worth mentioning here that the new results for the uppermost (VIII) are very comparable to previous determinations from charcoal and shell samples collected from analogous contexts during Tixier’s excavations in the 1970s. In fact, the charcoal from 8ac previously dated in Oxford (OxA-1798: 29300 ± 800 BP; [26]) produced a more precise but statistically identical date when treated with the ABOx-SC method (OxA-19194: 30250 ± 170 BP). This suggests that the start of Phase 5 can be placed at around 30 ka BP and most probably slightly earlier.

It is, therefore, only the middle part of the sequence (levels XX–XVII from 12.95 to 10.90 m below datum) that exhibits the largest chronometric variation. There is no technical reason to suggest that the outlying determinations are problematic, except in the few occasions when diagenetic alteration has been detected. The reasons for variations in age must therefore lie elsewhere.

It was expected that due to the large spits (artificial excavation units), the substantial geological layers the material was excavated from and the overall very deep sequence the effects of severe mixing of material during these early excavations should be minimal; some of the dates, however, reveal different problems: the possibility of post-excavation mixing or of secondary deposition of the material or disassociation with the context they are supposed to date.

With regards to post-excavation mixing, the discrepancy of the dates for 5 *Columbella rustica* shells is a case in point. When directly dated these shells yielded consistently younger dates than other species from the same contexts: three samples from levels XXII and XVII (Phases 1 and 2) were dated between 33–31 ka BP, an age span more consistent with the upper parts of the sequence (Phases 3-6) which should have been firmly separated by the cemented Stone Complex 2 by more than 7 m of deposit. We ought to point out here that the material as curated today at the Naturalis (previously at a University Museum Collection) is categorized by genus/species, then grouped on the basis of layers and sometimes of similar squares and depth assignation. This classification certainly happened post-excavation and mistakes or mixing of material cannot be excluded.

With regards to the accuracy of level assignation, as it has been already pointed out Ewing’s excavation was conducted and was based on geological layers which do not necessarily align to the archaeological/cultural levels and occupation horizons [8]. The lack of detailed excavation notes to account for differences between squares (e.g. sloping of the sediments) and to describe the actual association of levels or specific contexts with rockfalls, sedimentary hiatuses and brecciated deposits, pits, hearths and other structures affecting the deposition of each sample, render any attempt to explain the discrepancies somewhat speculative. In support of this, variations in the consistency of dates within squares are clearly evident. When only the shell dates from E 4 are plotted together, the model demonstrates adequate chrono-stratigraphic correlation (Fig. S3), which is not the case with square F 4. The burial(s) found in levels (XVI/ XVII/ XVIII) in square F 3 at the back of the cave, may have caused significant disturbance of the surrounding deposit, especially in neighbouring square F 4. This may have occurred in antiquity during the deposition of the human remains but also during the recovery of the skeleton(s). As mentioned by Ewing [33, 43] the skeleton of the child was found in 1938 directly capped by Complex 2 and was covered in a cast of cement only to be removed from the site ten years later when excavations resumed. Any hypotheses of sedimentary disturbance in the relevant areas are difficult to test without examining the site itself and all types of material coming from the squares and depths in question. We ought to note, however, that Ewing emphasized in several publications [14, 43] the hardness of the sediment, especially when in the proximity of the Stone complexes, which must have rendered meticulous excavation, accurate recovery and placement of the artifacts in their exact context rather difficult.

Despite the aforementioned problems the new determinations represent a significant step forward in the dating of this key Levantine site.

**Section III:**

**Bayesian-based chronological framework**

The application of statistical methods in the treatment of radiocarbon dates has become increasingly important over the last two decades. Bayesian statistical methods allow archaeological “prior” information derived from the overall sequence, as well as more specific contexts, to be incorporated into a formal model framework [44]. An introduction to Bayesian modelling in archaeology is provided by Buck et al. [45].

The radiocarbon dataset from Ksar Akil was incorporated into a Bayesian statistical model built with OxCal 4.1.7 [46]. The archaeological information regarding the location of each sample was used as ‘prior information’ to place each date in specific phases representing archaeological episodes. OxCal produces a new series of probability distributions (‘posterior’ distributions) and determines probabilistically the degree of agreement between single calibrated determinations and the overall sequence. The robustness of the model and any aberrant results are assessed using agreement indices and outlier detection analysis [47]. Here we used a t-type outlier model with prior probability of 0.05 for the new dates (5% chances that the date is aberrant), but we entered higher prior probabilities (0.1-0.5) to downweigh previous measurements produced through less refined pretreatment methods. By using boundaries (undated events) constraining the start and end of each phase of the model, we obtain probability distributions which are of key importance in understanding transition events; the beginnings and ends of the archaeological phases of interest.

Initially, 39 dates (27 new, plus 12 old determinations from Ewing’s Mousterian levels and Tixier’s late UP phases) were incorporated in a model structured around the individual levels from which the samples dated derive (Fig. S4), and outlier detection analysis was performed (Model 1). Twenty-six of these dates are on marine shell (OxA numbers above 20000s and OxA-Xs), one on landsnail (GrN-2195), eight on charcoal (OxA-19194, OxA-1797-1805, MC-1191-1192), two on clay treated as charcoal (GrN-2579 and Gro-2574/75; possibly referring to the same sample) and 2 U-series dates on bone. In this initial modeling attempt, 11 of the 39 determinations were identified as outliers. The problematic section of the model relates to layers XXII, XVIII and XVII.

In a second model (Model 2), the same 39 determinations were included but this time the constituent levels were combined within five broad techno-typologically distinct phases: Mousterian, Ksar Akil Phase 1, Phase 2 and so on (Fig. S5). Within the individual phases, the assigned depth for each shell was not taken into account since in reality this only refers to the topmost depth of the archaeological spit (sometimes 1m deep) and not the actual depth the material was found at. These broad phases represent large, ~2 m thick stratigraphic slices. In this case, the model was more robust, fewer outliers were identified (9 of 39). Four of these (OxA-25655, OxA-25652, OxA-X-2361-14, OxA-X-2342-57) are dates on *Columbella rustica* shells, and a younger one (GrN-2195) on landsnail(s) which come from dubious context (open section, 6-7.5 m). The two remaining dates (OxA-20880, OxA-20667) are also young for their context, but it should be noted that they are compared to determinations of shells from different squares of the site for which detailed slope and other stratigraphic information is not available.

The output of the two models is very similar and only slightly differences are observed, mainly at the duration of the Early Ahmarian and the beginning of the Late Upper Palaeolithic phases (see Main Text). The number of outliers, ~23%, remains elevated but the shell beads of *C. rustica* are mainly responsible for it. If these are excluded the number of outliers drops to about 12%.

At present this is the most reliable information we can deduce from the existing determinations and we hope that the new chronometric sequence for Ksar Akil will enable researchers draw direct comparisons with this reference Upper Palaeolithic site, beyond the limitations of lithic techno-typological similarities and “*fossiles directeurs*”.

**REFERENCES**

1. Azoury I (1986) Ksar Akil, Lebanon: A technological and typological analysis of the transitional and Early Upper Palaeolithic levels at Ksar Akil and Abu Halka. Oxford, BAR International Series 289 (i and ii).
2. Murphy JW (1938) The Method of Pre-historic Excavations at Ksar ‘Akil. Anthropology Series, Boston College Graduate School 3: 211-275.
3. Murphy JW (1939) Ksar ‘Akil, Boston College Expedition. Anthropology Series, Boston College Graduate School 3: 211-217.
4. Bergman CA (1981) Point Types in the Upper Palaeolithic Sequence at Ksar Akil, Lebanon. In: Cauvin J, Sanlaville P, editors. Préhistoire du Levant: chronologie et organisation de l’espace depuis les ori- gines jusqu’au VIe millénaire. Colloque international du CNRS, Lyon, Maison de l’Orient méditerranéen, 10-14 juin 1980. Paris: Éditions du CNRS (Colloques internationaux du CNRS 598). pp. 319-330.
5. Bergman CA (1987) Ksar Akil, Lebanon: A Technological and Typological Analysis of the Later Paleolithic Levels of Ksar Akil. Volume II: Levels XIII-VI. Oxford, BAR International Series 329.
6. Copeland L (1987) Preface. In: Bergman CA. Ksar Akil, Lebanon: A Technological and Typological Analysis of the Later Paleolithic Levels of Ksar Akil. Volume II: Levels XIII-VI. Oxford: BAR International Series 329, pp iv-ix.
7. Tixier J, Inizan M-L (1981) Ksar Aqil, stratigraphie et ensembles lithiques dans le Paléolithique supérieur: fouilles 1971-1975. In: Cauvin J, Sanlaville P, editors. Préhistoire du Levant: chronologie et organisation de l’espace depuis les ori- gines jusqu’au VIe millénaire. Colloque international du CNRS, Lyon, Maison de l’Orient méditerranéen, 10-14 juin 1980. Paris: Éditions du CNRS (Colloques internationaux du CNRS 598). pp. 353-367.
8. Williams JK, Bergman CA (2010) Upper Paleolithic Levels XIII-VI (A and B) from the 1937-1938 and 1947-1948 Boston College Excavations and the Levantine Aurignacian at Ksar Akil, Lebanon. Paléorient 36: 117-161.
9. Williams JK (2003) Examining the Boundaries of the Levantine Aurignacian. Unpublished Ph.D. dissertation. Dallas: Southern Methodist University.
10. Tixier J (1970) L’Abri sous roche de Ksar ’Aqil. La campagne de fouilles 1969 [The rockshelter of Ksar Akil. The 1969 excavations]. Bulletin du Musée de Beyrouth 33:173-191. French
11. Tixier J (1974) Fouille à Ksar'Aqil, Liban (1969-1974) [Excavation at Ksar Akil, Lebanon (1969-1974)]. Paléorient 2:187-192. French
12. Wright, Jr, HE (1951) Ksâr ʾAkil: Its archeological sequence and geological setting. J Near East Stud 10:113-122.
13. Wright, Jr, HE (1961) Late Pleistocene Geology of Coastal Lebanon. Quaternaria 6:525-541.
14. Ewing JF (1948) Ksâr ‘Akil in 1948. Biblica 29: 272-278.
15. Marks AE, Volkman P (1986) The Mousterian of Ksar Akil: Levels XXVIA through XXVIIIB. Paléorient 12:5-20.
16. Marks AE (1983) The Middle to Upper Paleolithic Transition in the Levant. In: Wendorf F, Close AE, editor. Advances in World Archaeology, vol. 2. New York: Academic Press. pp. 51-92.
17. Ohnuma K, Bergman CA (1990) A Technological Analysis of the Upper Paleolithic Levels (XXV-VI) of Ksar Akil, Lebanon. In: Mellars P, editor. The emergence of modern humans: An archaeological perspective. Edinburgh: Edinburgh University Press. pp. 91-138.
18. Bergman CA (1988) The Upper Palaeolithic of the Levant. In: Aurenche O, Cauvin M-C, Sanlaville P, editors. Préhistoire du Levant II. Processus des changements culturels. Hommage à Francis Hours. Paléorient 14 (2): 223-227.
19. Kuhn S, Stiner MC, Kerry KW, Güleç E (2003) The Early Upper Palaeolithic at Üçağızlı Cave (Hatay, Turkey): Some Preliminary Results. In: Goring-Morris AN, Belfer-Cohen A, editors. More than Meets the Eye: Studies on Upper Palaeolithic Diversity in the Near East. Oxford: Oxbow Books. pp. 106-117.
20. Kuhn S (2003) In What Sense is the Levantine Initial Upper Paleolithic a ‘‘Transitional’’ Industry? In: Zilhão J, d’Errico F, editors. The chronology of the Aurignacian and of the Transitional technocomplexes: Dating, Stratigraphies, Cultural Implications. Lisbon: Istituto Português de Arqueologia (Trabalhos de Arqueologia 33). pp 61-70.
21. Newcomer MH (1968-1969) The chamfered pieces from Ksar Akil (Lebanon). Bulletin of the Institute of Archaeology, London 8, 9:177-191.
22. Kuhn SL, Stiner MC, Güleç E., Özer I, Yilmaz H, et al. (2009) The early Upper Paleolithic occupations at Üçağızlı Cave (Hatay, Turkey). J Hum Evol 56: 87-113
23. Goring-Morris AN, Belfer-Cohen A (2006) A Hard Look at the “Levantine Aurignacian”: How Real is the Taxon? In: Bar-Yosef O, Zilhão J, editors. Towards a Definition of the Aurignacian. Lisbon: Instituto Português de Arqueologia (Trabalhos de Arqueologia 45). pp 297-314.
24. Chiotti L (2000) Lamelles Dufour et Grattoirs Aurignaciens (carénés et à museau) de la couche 8 de l’Abri Pataud, Les-Eyzies-de-Tayac, Dordogne [Aurignacian Dufour bladelets and scrapers (carinated and nosed) in layer 8 of Abri Pataud, Les-Eyzies-de-Tayac, Dordogne]. L’Anthropologie 104: 239-263. French
25. Hays MA, Lucas G (2000) A Technological and Functional Analysis of Carinates from Le Flageolet I, Dordogne, France. J Field Archaeol 27: 455-465.
26. Mellars P, Tixier J (1989) Radiocarbon-accelerator dating of Ksar Aqil (Lebanon) and the chronology of the Upper Paleolithic sequence in the Middle East. Antiquity 63: 761-768.
27. Vogel JC, Waterbolk HT (1963) Groningen radiocarbon dates IV. Radiocarbon 5:163-202.
28. Van der Plicht J, van der Wijk A, Bartstra GJ (1989) Uranium and thorium in fossil bones: activity ratios and dating. Appl Geochem 4: 339-342.
29. Brock F, Higham T, Bronk Ramsey T (2010) Pre-screening techniques for identification of samples suitable for radiocarbon dating of poorly preserved bones. J Archaeol Sci 37: 855-865
30. Douka k, Hedges REM, Higham TFG (2010) Improved AMS ^14^C dating of shell carbonates using high-precision X-Ray Diffraction (XRD) and a novel density separation protocol (CarDS). Radiocarbon 52: 735-751.
31. Altena Van Regteren CO (1962) Molluscs and Echinoderms from the Palaeolithic deposits in the rock shelter of Ksar ’Akil, Lebanon. Zoologische Mededelingen 38:87-99.
32. Inizan, M.L., Gaillard, J.M., 1978. Coquillages de Ksar-'Aqil : éléments de parure ? [Shells of Ksar Akil: Elements of ornamentation?] *Paléorient* 4:295-306. French
33. Ewing JF (1949) The Treasures of Ksar Akil. Thought 24: 255-288.
34. Kuhn SL, Stiner MC, Reese DS, Güleç E (2001) Ornaments in the earliest Upper Paleolithic: new results from the Levant. Proc Natl Acad Sci USA 98: 7641-7646.
35. Hooijer DA (1961) The fossil vertebrates of Ksar 'Akil, a Palaeolithic rock shelter in the Lebanon. Zoologische Verhandelingen 49:4-67.
36. Douka K (2011) An Upper Palaeolithic shell scraper from Ksar Akil (Lebanon). J Archaeol Sci 38:429-437.
37. Brock F, Higham TFG, Ditchﬁeld P, Ramsey CB (2010) Current pre-treatment methods for AMS radiocarbon dating at the Oxford Radiocarbon Accelerator Unit (ORAU). Radiocarbon 52:103-112.
38. Reimer PJ, Baillie MGL, Bard E, Bayliss A, Beck JW, et al (2009) IntCal09 and Marine09 radiocarbon age calibration curves, 0–50,000 years cal BP. Radiocarbon 51:1111-1150.
39. Reimer PJ, McCormac FG (2002) Marine radiocarbon reservoir corrections for the Mediterranean and Aegean Seas. Radicoarbon 44: 159-166.
40. Iovita RP (2009) Reevaluating connections between the Early Upper Palaeolithic of Northeast Africa and the Levant: technological differences between the Dabban and the Emiran. In: Shea J, Leiberman D, editors. Transitions in Prehistory: Papers in Honor of Ofer Bar-Yosef. Oxford: Oxbow Books for the American School of Prehistoric Research. pp.127-144.
41. Sivan D, Potasman M, Almogi-Labin A, Mayer Bar-Yosef DE, Spanier E, Boaretto E (2005) The *Glycymeris* query along the coasts and shallow shelf of Israel, southeast Mediterranean. Palaeogeogr Palaeoclimatol Palaeoecol 233: 134-148.
42. Mienis, H.K., Ben-David Zaslow, R., Bar-Yosef Mayer D.E. (2006) *Glycymeris* in the Levant Sea 1. Finds of recent *Glycymeris insubrica* in the south east corner of the Mediterranean sea. Triton 13: 5-9.
43. Ewing JF (1947) Preliminary Note on the Excavations at the Paleolithic Site of Ksar Akil, Republic of Lebanon. Antiquity 21:186-196.
44. Bronk Ramsey C (1998) Probability and dating. Radiocarbon 40: 461-474.
45. Buck CE, Cavanagh WG, Litton CD (1996) Bayesian approach to interpreting archaeological data. Chichester: Wiley.
46. Bronk Ramsey C (2009) Bayesian analysis of radiocarbon dates. Radiocarbon 51: 337-360.
47. Bronk Ramsey C (2009) Dealing with outliers and offsets in radiocarbon dating. Radiocarbon 51: 1023-1045.
